# Pretargeting of internalizing trastuzumab and cetuximab with a ^18^F-tetrazine tracer in xenograft models

**DOI:** 10.1186/s13550-017-0344-6

**Published:** 2017-12-02

**Authors:** Outi Keinänen, Kimberly Fung, Jacob Pourat, Vilma Jallinoja, Delphine Vivier, NagaVara Kishore Pillarsetty, Anu J. Airaksinen, Jason S. Lewis, Brian M. Zeglis, Mirkka Sarparanta

**Affiliations:** 1 Department of Chemistry, Radiochemistry, University of Helsinki, P.O. Box 55, FI-00014 Helsinki, Finland; 2 Department of Radiology, Memorial Sloan Kettering Cancer Center, 1275 York Avenue, New York, NY 10065 USA; 3 Department of Chemistry, Hunter College and the Graduate Center of the City University of New York, 695 Park Avenue, New York, NY 10065 USA; 4 Ph.D. Program in Chemistry, Graduate Center of the City University of New York, New York, 10016 NY USA; 5 Program in Molecular Pharmacology, Memorial Sloan Kettering Cancer Center, 1275 York Avenue, New York, NY 10065 USA; 6 Department of Radiology, Weill Cornell Medical College, 1300 York Avenue, New York, NY 10065 USA; 7 Department of Pharmacology, Weill Cornell Medical College, 1300 York Avenue, New York, NY 10065 USA

**Keywords:** Fluorine-18, Inverse electron-demand Diels-Alder (IEDDA) reaction, Pretargeting, Tetrazine, *Trans*-cyclooctene

## Abstract

**Background:**

Pretargeting-based approaches are being investigated for radioimmunoimaging and therapy applications to reduce the effective radiation burden to the patient. To date, only a few studies have used short-lived radioisotopes for pretargeting of antibodies, and such examples with internalizing antibodies are even rarer. Herein, we have investigated pretargeting methodology using inverse electron-demand Diels-Alder (IEDDA) for tracing two clinically relevant, internalizing monoclonal antibodies, cetuximab and trastuzumab.

**Results:**

Bioorthogonal reaction between tetrazine and *trans*-cyclooctene (TCO) was used for tracing cetuximab and trastuzumab in vivo with a fluorine-18 (*t*_½_ = 109.8 min) labelled tracer. TCO-cetuximab or TCO-trastuzumab was administered 24, 48, or 72 h prior to the injection of tracer to A431 or BT-474 tumour-bearing mice, respectively. With cetuximab, the highest tumour-to-blood ratios were achieved when the lag time between antibody and tracer injections was 72 h. With trastuzumab, no difference was observed between different lag times. For both antibodies, the tumour could be clearly visualized in the PET images with the highest tumour uptake of 3.7 ± 0.1%ID/g for cetuximab and 1.5 ± 0.1%ID/g for trastuzumab as quantified by ex vivo biodistribution. In vivo IEDDA reaction was observed in the blood for both antibodies, but with trastuzumab, this was to a much lower degree than with cetuximab.

**Conclusions:**

We could successfully visualize the tumours by using cetuximab and trastuzumab in pretargeted PET imaging despite the challenging circumstances where the antibody is internalized and there is still some unbound antibody circulating in the blood flow. This clearly demonstrates the potential of a pretargeted approach for targeting internalizing antigens and warrants development of pharmacokinetic optimization of the biorthogonal reactants to this end.

**Electronic supplementary material:**

The online version of this article (10.1186/s13550-017-0344-6) contains supplementary material, which is available to authorized users.

## Background

**Fig. 1:**
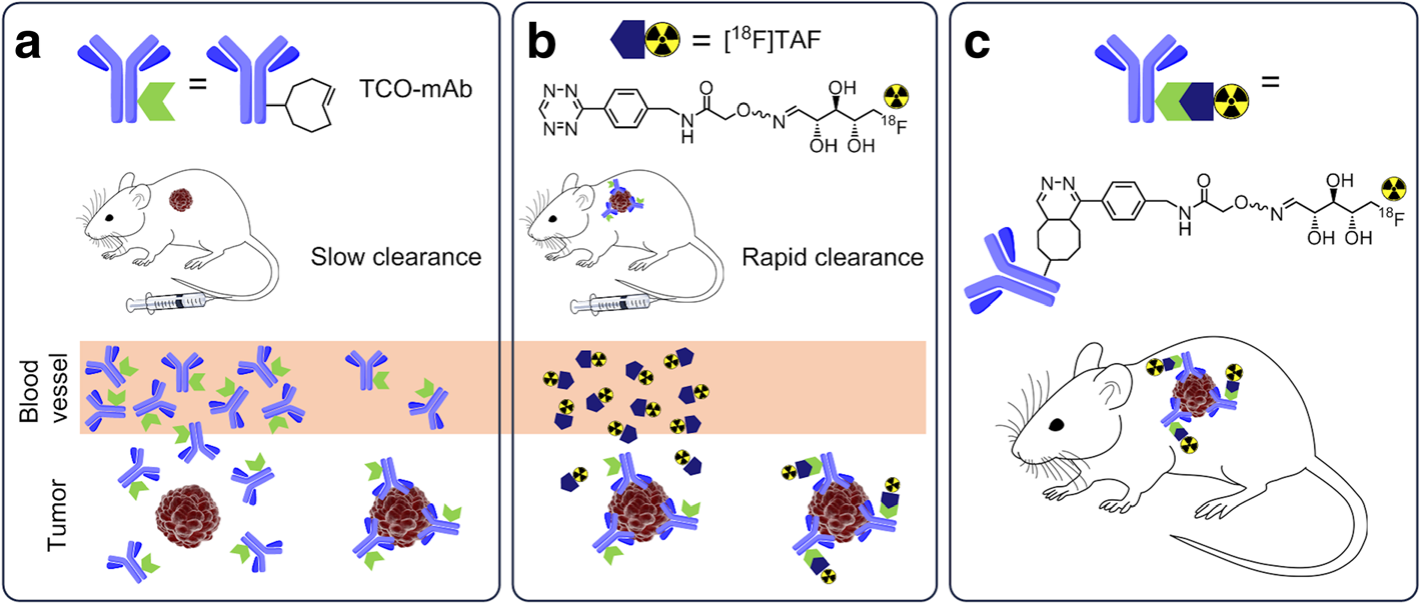
Conceptual representation of a two-step pretargeted method based on IEDDA reaction. The current approach involves the sequential administration of an antibody, followed by administration of a small molecule radiotracer after 24–72 h. **a** TCO-modified antibody is administered first. It circulates for a couple of days and eventually accumulates in the tumour and clears from the blood pool and other non-target tissues. **b** After a predetermined lag time, a radiolabelled tetrazine tracer is administered. It reacts selectively with the TCO group attached to the antibody and the unreacted tracer clears rapidly from the blood. In this work, the used radiotracer was ^18^F-labelled tetrazine, [^18^F]TAF. **c** The antibody can now be localized via the radiolabel on the tracer. The pretargeting approach with short-lived tracer significantly lowers the radiation burden to the subject compared to traditional methods where the antibody is directly radiolabelled with a longer-lived radionuclide

During recent years, the radiolabelling of clinically relevant monoclonal antibodies (mAbs), like cetuximab and trastuzumab, has been under intense investigation for the generation of diagnostic and therapeutic radiopharmaceuticals that can identify and treat tumours expressing the target antigen [1]. Traditional radiolabelling methods of mAbs for PET (positron emission tomography) and SPECT (single-photon emission computed tomography) usually involve the use of long-lived radioisotopes, such as zirconium-89 (*t*_½_ = 3.3 days), indium-111 (*t*_½_ = 2.8 days), and iodine-124 (*t*_½_ = 4.2 days); the half-lives of which are matched to the biological half-lives of mAbs. Although the target-to-background ratios obtained with directly radiolabelled mAbs are excellent, typically, several hours to days are required before non-target radioactivity has decreased to sufficiently low levels necessitating the use of long-lived isotopes. An additional caveat is the increased radiation burden to the subject, especially to the bone marrow resulting from long circulation time of mAbs. In pretargeting, the antibody is radiolabelled in vivo after it has accumulated to the tumour and most of the unbound antibody has cleared from the blood flow (Fig. 1), enabling the use of short-lived radioisotopes with dosimetric benefits [2–4]. Earlier approaches to pretargeting included streptavidin-biotin system [5], bispecific antibodies targeting the antigen with In-DTPA [6] or Y-DOTA [7] complexes, and antibody-modified oligonucleotide conjugates [8]. In the recent years, bioorthogonal chemical reactions have emerged as efficient tools for pretargeted nuclear imaging of mAbs [2, 4, 9–15]. Bioorthogonal reactions occur under physiological conditions without interfering with or being interfered by any of the native biochemical processes of the system [16, 17]. The most studied bioorthogonal reactions for pretargeting are the strain-promoted alkyne azide cycloaddition (SPAAC) and the inverse electron-demand Diels-Alder (IEDDA) cycloaddition between a tetrazine and a dienophile.

The radioisotopes used in previous in vivo pretargeting studies have included indium-111 (*t*_½_ = 2.8 days) [15, 18], copper-64 (*t*_½_ = 12.7 h) [2, 13, 19], technetium-99 m (*t*_½_ = 6.0 h) [12, 20], fluorine-18 (*t*_½_ = 109.8 min) [4, 21, 22], gallium-68 (*t*_½_ = 67.6 min) [11], and carbon-11 (*t*_½_ = 20.4 min) [23]. The half-life dictates the maximum time allowed after tracer injection for imaging. With indium-111, copper-64, and technetium-99 m, the time needed for the clearance of the tracer from non-target tissues plays a lesser role because the imaging can be performed several hours or even days later than the tracer injection. While the advantage in the use of the latter short-lived radioisotopes is that the effective dose to the subject is decreased, the challenge is to modify the pharmacokinetics of the tracer to allow for rapid elimination within the imaging time frame dictated by the shorter half-life. High molar activity ^18^F is nowadays widely available in clinical PET centres, and the half-life of 109.8 min of ^18^F allows for multistep synthesis, the preparation of multiple imaging doses from one batch of the radiotracer, and transportation of the tracer to remote sites if needed. Therefore, the choice of a fluorine-18-labelled tracer balances between the dosimetric limitations in the use of the long-lived radiometal-labelled tracers and the synthetic challenges of short-lived ^68^Ga- and ^11^C-labelled ones. In this work, we used a previously developed ^18^F-radiolabelled tetrazine tracer, [^18^F]TAF [24] (Fig. 1). The radiosynthesis of [^18^F]TAF is carried out using [^18^F]-5-fluoro-5-deoxy-ribose ([^18^F]FDR) as a prosthetic group to radiolabel aminooxy-functionalized tetrazine via oxime formation. The fluoroglycosylation with [^18^F]FDR is a very attractive choice for radiolabelling tetrazines with fluorine-18 since it is a high-yield reaction with mild reaction conditions compatible with the inherent instability of tetrazines. In addition, by using different linkers between the tetrazine and the aminooxy group, the structure of the tetrazine radiotracer can be altered depending on the application. We have previously used [^18^F]TAF successfully for pretargeting of porous silicon (PSi) nanoparticles after intravenous administration [25]. Although radiotracers labelled with longer-lived radioisotopes including ^64^Cu and ^111^In have been successfully used for pretargeting antibodies previously [9, 15], the time between the injection of the radiotracer and the start of the imaging acquisition have been rather long, and this lag time as well as the radioburden to the subject could be decreased further with radiotracers labelled with shorter-lived isotopes.

We have previously shown that the higher the molar activity of the tetrazine radiotracer, the more efficient the bioorthogonal reaction is in vivo [25], while others view that diluting the tetrazine radiotracer with additional non-radiolabelled compound is necessary for maintaining high reaction rates in vivo [4, 9, 26]. Since our earlier experiments were done with nanoparticles that were cleared from the blood rapidly, we selected an amount of 1:1 ratio of cold TAF tetrazine to the amount of *trans*-cyclooctene (TCO) in the antibody because this has been shown to be successful in the literature specifically in antibody pretargeting. This approach also provides both blocking of the excess TCO in the blood as well as driving of the equilibrium of the bioorthogonal reaction towards the product.

Herein, we have investigated the efficacy of two-step pretargeting strategy based on IEDDA reaction for tracing two clinically relevant antibodies, cetuximab and trastuzumab, with [^18^F]TAF (Fig. 1) in epidermal growth factor receptor (EGFR)-positive (A431 epidermoid carcinoma) and HER2-positive (BT-474 ductal carcinoma) xenografts in vivo. Cetuximab binds to the extracellular domain of the EGFR that is over-expressed and/or activated in many types of cancer. Trastuzumab is an anti-HER2 mAb that is used for the treatment of HER2/*neu*-positive breast cancers. Both EGFR (ErbB1/HER1) and HER2/*neu* (ErbB2) belong to the HER family of tyrosine kinase receptors. Both cetuximab and trastuzumab exhibit slow pharmacokinetics and are known to internalize over time [27–31]. In principle, the cell membrane-bound antibody is the only fraction that is available for pretargeting with a non-internalizing radiotracer. Notably, the antibodies previously used successfully for pretargeted imaging either accumulate to the tumour at exceptionally high concentrations such as 5B1 targeting the carbohydrate antigen CA 19.9 in pancreatic cancer [32, 33] or show high persistence on the cell surface even when bound to the antigen, like huA33 targeting a transmembrane glycoprotein in colorectal cancer [9, 34]. In this work, the known internalization of the selected mAbs brings an additional challenge for the pretargeted system together with the residual unbound antibody remaining in the blood flow at the time of tracer injection. Despite these complications, we could localize the tumour in the PET images, showing that pretargeted imaging strategies could be extended to the imaging of a wider selection of antibodies with careful tailoring of the radiotracer pharmacokinetics.

## Methods

### Cell lines

Human tumour cell lines A431 (epidermoid carcinoma) and BT-474 (ductal carcinoma) were obtained from the American Tissue Culture Collection (ATCC, Manassas, VA, USA). A431 cells were cultured in DMEM supplemented with 10% fetal bovine serum, 4 mM l-glutamine, 4.5 g/l glucose, 1 mM sodium pyruvate, 1.5 g/l sodium bicarbonate, penicillin, and streptomycin. BT-474 cells were cultured in DMEM:F-12 (1:1) supplemented with 10% fetal bovine serum, 2 mM l-glutamine, nonessential amino acids, penicillin, and streptomycin. All media were purchased from the Media Preparation Facility at MSKCC (Memorial Sloan Kettering Cancer Center, New York, NY, USA). Cells were maintained in atmosphere containing 5% CO_2_ at 37 °C. Cells were harvested and passaged weekly using 0.25% trypsin/0.53 mM EDTA in HBSS without calcium or magnesium. See Additional file 1 (section “General Materials and Methods”) for the information on A549 and SKOV3 cell lines that were used in cell studies.

### Antibodies

Multidose formulations of cetuximab were obtained from ImClone (Erbitux), trastuzumab was obtained from Genentech (Herceptin), and immunoglobulin G (IgG) was obtained from Fisher Scientific (Human IgG, Whole Molecule Control, Invitrogen). Trastuzumab was purified before use to remove α-α-trehalose dehydrate, l-histidine, and polysorbate 20 additives. Cetuximab was also treated analogously before use, although the formulation did not include any possibly interfering ingredients. The purification was done with three altering steps of size-exclusion chromatography (PD10, GE Healthcare Life Sciences, Little Chalfont, Buckinghamshire, UK) and with spin-column centrifugation (Ultracel-50, Amicon, EMD Millipore, Billerica, MA, USA).

### DFO-modification of mAbs

Cetuximab was conjugated to desferrioxamine B-p-SCN (DFO-p-SCN in DMSO, 5 eq) under mildly basic conditions (pH 8.5–8.7). The mixture was incubated for 1 h at 37 °C and shaken at 500 rpm. DFO-cetuximab was purified with size-exclusion chromatography (PD10) and with spin-column centrifugation three times (Ultracel-50, Amicon, 50 kD cutoff). MALDI mass spectrometry was used to determine the number of DFO moieties that had been added to each molecule of cetuximab. DFO-IgG was prepared in a manner similar to DFO-cetuximab. Readily conjugated DFO-trastuzumab was a kind gift from the Radiochemistry & Molecular Imaging Probes Core Facility at MSKCC.

### ^89^Zr-labelling of DFO-mAbs and the isotype IgG control

Zirconium-89 was produced through proton beam bombardment of yttrium foil and isolated in high purity as ^89^Zr-oxalate at MSKCC according to a previously established procedure [35]. [^89^Zr]Zr-DFO-cetuximab was prepared by the complexation of ^89^Zr-oxalate with DFO-cetuximab. ^89^Zr-oxalate (114.7 MBq) in 1.0 M oxalic acid (80 μl) was adjusted to pH 7.1–7.5 with 1.0 M Na_2_CO_3_. After CO_2_ evolution ceased, DFO-cetuximab (0.756 mg in 250 μl sterile Chelex-treated PBS) was added, and the reaction was mixed gently by aspirating with a pipette. The reaction mixture was incubated at 37 °C for 1 h. [^89^Zr]Zr-DFO-cetuximab was purified with size-exclusion chromatography (PD10) and concentrated with spin-column centrifugation (Ultracel-50, Amicon). [^89^Zr]Zr-DFO-trastuzumab and [^89^Zr]Zr-DFO-IgG were prepared in a similar manner as [^89^Zr]Zr-DFO-cetuximab.

### TCO-modification of mAbs and the isotype IgG control

An activated succinimidyl ester of TCO ((*E*)-cyclooct-4-en-1-yl (2,5-dioxopyrrolidin-1-yl) carbonate in DMSO, 40 eq) was incubated with mAb for 1 h at RT at pH 8.5–8.7 and shaken 500 rpm. TCO-mAb was purified with size-exclusion chromatography (PD10) and with spin-column centrifugation three times (Ultracel-50). The resulting modified mAbs were characterized by MALDI mass spectrometry.

### Compound TAF and radiosynthesis of [^18^F]TAF

No-carrier-added [^18^F]fluoride was produced in a ^18^O(p,n)^18^F nuclear reaction on a GE Healthcare PET Trace 800 cyclotron using 16.5 MeV protons and enriched ^18^O-water. Compound TAF, [^18^F]TAF (see Additional file 1: Scheme S1) and its precursors were synthesized as previously described [24]. Cell uptake assay was performed to ensure that [^18^F]TAF has no cell uptake on its own (see experimental details from Additional file 1, section 3).

### Animal experiments

All animal experiments were carried out in compliance with protocol approved by the Institutional Animal Care and Use Committee of Memorial Sloan Kettering Cancer Center and followed National Institutes of Health guidelines for animal welfare. Animals were group housed with water and food ad libitum. Mice were anaesthetized with 2% isoflurane/medical air inhalation and 5 × 10^6^ (A431) or 5–10 × 10^6^ (BT-474) cells were inoculated subcutaneously into the shoulder of 8–10 week-old female athymic *nu*/*nu* nude mice (Charles River, Wilmington, MA, USA) in a 150 μl suspension of 1:1 fresh media/Matrigel. For BT-474 tumour-bearing mice 17-β-estradiol pellets (0.72 mg/60-day release, Innovative Research of America, Sarasota, FL, USA) were surgically implanted subcutaneously under isoflurane anaesthesia 6 days before the cells were inoculated, and the skin was closed with a single wound clip. The animals received a single injection of meloxicam (2.0 mg/kg in sterile saline subcutaneously) for perioperative pain control. After 2 weeks from A431 inoculation and 5 to 7 weeks from BT-474 inoculation, the appropriate size for experiments (5–10 mm in the largest diameter) of the tumours was achieved. For all intravenous injections, conscious mice were gently warmed with a heat lamp and placed on a restrainer. The tail was sterilized with alcohol pads, and injection took place via the lateral tail vein. The animals were sacrificed with CO_2_ asphyxiation followed by cervical dislocation at designated time points after tracer administration, and samples of tissues and body fluids were collected for radioactivity measurements and weighing. Tissue samples were counted on an automated gamma counter (Wizard^2^ 3″, PerkinElmer), and radioactivity decay correction was applied. The ex vivo biodistribution results were expressed as percentage of injected radioactivity dose per gram of tissue (%ID/g).

### [^89^Zr]Zr-DFO-mAb and isotype IgG control studies

[^89^Zr]Zr-DFO-cetuximab (12.1 ± 1.1 MBq, 75 μg, in PBS; *n* = 4), [^89^Zr]Zr-DFO-trastuzumab (7.4 ± 0.1 MBq, 20 μg, in PBS; *n* = 3), [^89^Zr]Zr-DFO-IgG in A431 tumour model (9.0 ± 0.3 MBq, 75 μg, in PBS; *n* = 3), and [^89^Zr]Zr-DFO-IgG in BT-474 tumour model (4.1 ± 0.2 MBq, 20 μg, in PBS, *n* = 3) were administered intravenously. PET imaging was performed at 24, 48, 72, 96, and 120 h after injection of tracer.

### Pretargeted experiments

#### Cetuximab

In the pretargeted cetuximab experiments for group A (*n* = 4), TCO-cetuximab (75 μg, 0.5 nmol, 3.1 nmol of TCO, in PBS) was administered 24, 48, or 72 h prior to the injection of [^18^F]TAF (16.3 ± 1.8 MBq, 4.3 ± 0.1 nmol, in 10% EtOH in PBS) to A431 tumour-bearing mice. In group B (*n* = 4), non-radioactive TAF (3.1 nmol, in PBS) was injected 5 min prior to the injection of [^18^F]TAF (17.8 ± 1.7 MBq, 1.2 ± 0.1 nmol, in 10% EtOH in PBS); otherwise, the experiments were performed as in group A. Two blocking studies were performed in order to ensure the specificity of the binding at the tumour site. First, TCO-cetuximab (75 μg, 0.5 nmol, 3.1 nmol of TCO, in PBS) was administered 72 h prior to the injection of [^18^F]TAF (11.1 ± 1.3 MBq, 2.68 ± 0.05 μmol, in 10% EtOH in PBS) to A431 tumour-bearing mice (*n* = 3). Second, TCO-cetuximab (75 μg, 0.5 nmol, 3.1 nmol of TCO, in PBS) and cetuximab (1875 μg, in PBS; 25-fold excess to TCO-cetuximab) was administered 72 h prior to the injection of [^18^F]TAF (8.8 ± 1.4 MBq, 4.3 ± 0.1 μmol, in 10% EtOH in PBS) to A431 tumour-bearing mice (*n* = 3).

#### Trastuzumab

With pretargeted trastuzumab in group A (*n* = 4), TCO-trastuzumab (20 μg, 0.13 nmol, 0.65 nmol of TCO, in PBS) was administered 48 h or 72 h prior to the injection of [^18^F]TAF (20.1 ± 1.9 MBq, 1.4 ± 0.1 nmol, in 10% EtOH in PBS) to BT-474 tumour-bearing mice. In group B (*n* = 4), non-radioactive TAF (0.65 nmol, in PBS) was injected 5 min prior to the injection of [^18^F]TAF (22.5 ± 2.7 MBq, 0.6 ± 0.1 nmol, in 10% EtOH in PBS); otherwise, the experiments were performed as in group A. Two blocking studies were performed in order to ensure the specificity of the binding at the tumour site. First, TCO-trastuzumab (20 μg, 0.13 nmol, 0.65 nmol of TCO, in PBS) was administered 72 h prior to the injection of [^18^F]TAF (10.3 ± 0.4 MBq, 0.52 ± 0.01 μmol, in 10% EtOH in PBS) to BT-474 tumour-bearing mice (*n* = 3). Second, TCO-trastuzumab (20 μg, 0.13 nmol, 0.65 nmol of TCO, in PBS) and trastuzumab (500 μg, in PBS, 25-fold excess to TCO-trastuzumab) were administered 72 h prior to the injection of [^18^F]TAF (8.8 ± 1.4 MBq, 4.3 ± 0.1 μmol, in 10% EtOH in PBS) to BT-474 tumour-bearing mice (*n* = 5).

#### Human IgG isotype control

In the pretargeted IgG experiments, TCO-IgG (75 μg, 0.5 nmol, 1.2 nmol of TCO, in PBS) was administered 72 h prior to the injection of [^18^F]TAF (12.8 ± 1.2 MBq, in 10% EtOH in PBS) to A431 tumour-bearing mice (*n* = 5), and TCO-IgG (20 μg, 0.13 nmol, 0.32 nmol of TCO, in PBS) was administered 72 h prior to the injection of [^18^F]TAF (13.7 ± 0.9 MBq, in 10% EtOH in PBS) to BT-474 tumour-bearing mice (*n* = 5).

#### [^18^F]TAF alone

Additional control animals received only [^18^F]TAF (14.2 ± 1.8 MBq, 0.7 ± 0.1 nmol, in 10% EtOH in PBS, *n* = 4).

### PET imaging

PET imaging was performed on a microPET Focus 120 dedicated small-animal scanner (Siemens Medical Solutions, Malvern, PA, USA). Mice were anaesthetized with 2% isoflurane/medical air inhalation approximately 5 min prior to recording the PET images and kept under anaesthesia during the PET scan. The energy and coincidence timing windows were 350–750 keV and 6 ns, respectively. Data was acquired as static images with a minimum of 20 million coincident events. Data were sorted into two-dimensional histograms by Fourier re-binning, and transverse images were reconstructed by filtered back-projection (FBP) into a 128 × 128 × 63 (0.72 × 0.72 × 1.3 mm) matrix. The image data were normalized to correct for non-uniformity of response of the PET, dead-time count losses, positron branching ratio, and physical decay to the time of injection, but no attenuation, scatter, or partial-volume averaging correction was applied. The measured reconstructed spatial resolution for the Focus 120 is approximately 1.6 mm in full width at half-maximum at the centre of the field of view. The counting rates in the reconstructed images were converted to percent of injected dose per weight (%ID/g) by use of a system calibration factor derived from the imaging of a mouse-sized water-equivalent phantom containing ^18^F. Images were analysed by using ASIPro VM software (Concorde Microsystems).

### Statistical analysis

Results of the assays were expressed as mean ± standard deviation of at least three independent experiments. Two-tailed paired *t* test was used to evaluate the statistical significance, and the probability of *p < 0.05 and **p < 0.005 were considered statistically significant. The statistical analysis was carried out using IBM SPSS Statistics 22 (IBM Corporation, Armonk, NY, USA) software.

## Results

### [^89^Zr]Zr-DFO-cetuximab, [^89^Zr]Zr-DFO-trastuzumab and [^89^Zr]Zr-DFO-IgG

The immunoreactive fraction of the DFO-cetuximab was found to be 84.2 ± 1.6% (*n* = 5) (see Additional file 1, section 4). On average 1.84 ± 0.17 DFO moieties had been attached to each molecule of cetuximab. On average, there were 1.03 DFO moieties per trastuzumab and the immunoreactivity of DFO-trastuzumab varied between 86 and 97%. On average, 1.32 ± 0.25 DFO moieties had been attached to each molecule of IgG. Crude radiolabelling yields and radiochemical purities (ITLC: 50 mM EDTA pH 5) were > 99%. The final radiochemical purities were > 99% and radiochemical yields were 76.3%, 79.4%, 82.3%, and 45.8% for [^89^Zr]Zr-DFO-cetuximab, [^89^Zr]Zr-DFO-trastuzumab, [^89^Zr]Zr-DFO-IgG (A431 experiments), and [^89^Zr]Zr-DFO-IgG (BT-474 experiments), respectively.

### TCO-cetuximab, TCO-trastuzumab and TCO-IgG

An average 6.1 ± 0.1, 4.9 ± 0.1, and 2.43 ± 0.1 TCO moieties had been added to each molecule of cetuximab, trastuzumab, and IgG, respectively. The immunoreactive fraction (see Additional file 1, section 4) using in vitro conjugated [^18^F]TAF was found to be 93.9 ± 1.0% (*n* = 5) and 90.8 ± 1.9% (*n* = 5) for TCO-cetuximab and TCO-trastuzumab, respectively. The immunoreactivities of the TCO-modified mAbs were higher than those of the DFO-modified mAbs, although there were more TCO moieties (6.1 and 4.9) than DFO moieties (1.8 and 1.03) attached per mAb. The higher molecular weight and more hydrophobic nature of DFO appears to have a slight impact on the number of moieties conjugated, as well as the immunoreactivity of the mAbs compared to TCO.

### [^18^F]TAF

The final radiochemical purity was > 99%, the decay-corrected yield was 62.3 ± 11.3% (*n* = 5), and the molar activity varied between 15 and 38 GBq/μmol. The cell uptake of [^18^F]TAF was < 1% during the 4 h of observation in the A549 (EGFR+), BT-474 (HER/*neu+*), and SKOV3 (HER/*neu+*) cell lines (see Additional file 1: Figure S1).

### Determination of optimal imaging time point for the pretargeting experiments with ^89^Zr-labelled antibodies

**Fig. 2:**
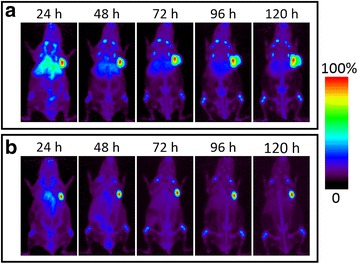
Maximum intensity projections (MIP) for representative mice administered with 11.4 MBq of [^89^Zr]Zr-DFO-cetuximab (**a**) and 7.3 MBq of [^89^Zr]Zr-DFO-trastuzumab (**b**) at different time points after injection in A431(**a**) and BT-474 (**b**) tumour-bearing mice (*n* = 3), respectively. The maximum intensity projections (MIPs) are scaled to the same percentages (100%) for intensity minimum and maximum to appropriately compare the images

DFO-cetuximab and DFO-trastuzumab were radiolabelled with ^89^Zr to visualize their biodistribution in A431 and BT-474 xenograft-bearing mice in order to determine the optimal time point (i.e., the highest tumour accumulation of the mAb) based on maximal tumour-to-background signal ratio for the pretargeted imaging experiment. The maximum intensity projections (MIP) of PET images of [^89^Zr]Zr-DFO-cetuximab and [^89^Zr]Zr-DFO-trastuzumab at different time points after injection are presented in Fig. 2. Coronal, sagittal, and transverse planar PET images that intersect the centre of the tumours and quantified ex vivo biodistribution of [^89^Zr]Zr-trastuzumab and [^89^Zr]Zr-cetuximab are shown in Additional file 1: Figure S2 and Table S1. Our data were in good agreement with previously published data on ^89^Zr-labelled trastuzumab and cetuximab [36–40]. The antibodies accumulated to the tumour site rapidly, but clearance from non-target tissues was seen only after 72 h, and the hepatobiliary elimination was more prominent and persisted longer for cetuximab. However, due to the known internalization of the used antibodies that subsequently lowers the surface-residing fraction available for the IEDDA reaction, time points earlier than 72 h were also investigated in the pretargeting experiment in order to attain optimal balance between the amount of surface-bound and freely circulating mAb.

### Pretargeting experiments

In vitro pretargeting was performed in order to estimate the efficiency of pretargeting TCO-cetuximab and TCO-trastuzumab with [^18^F]TAF. The binding of [^18^F]TAF to TCO-antibody-pretreated cells exceeded the nonspecific uptake of the ^18^F-labelelled tetrazine tracer alone over 75-fold, 20-fold, and 15-fold in BT-474, SKOV3, and A549 cells, respectively (see Additional file 1: Figure S4).

**Fig. 3:**
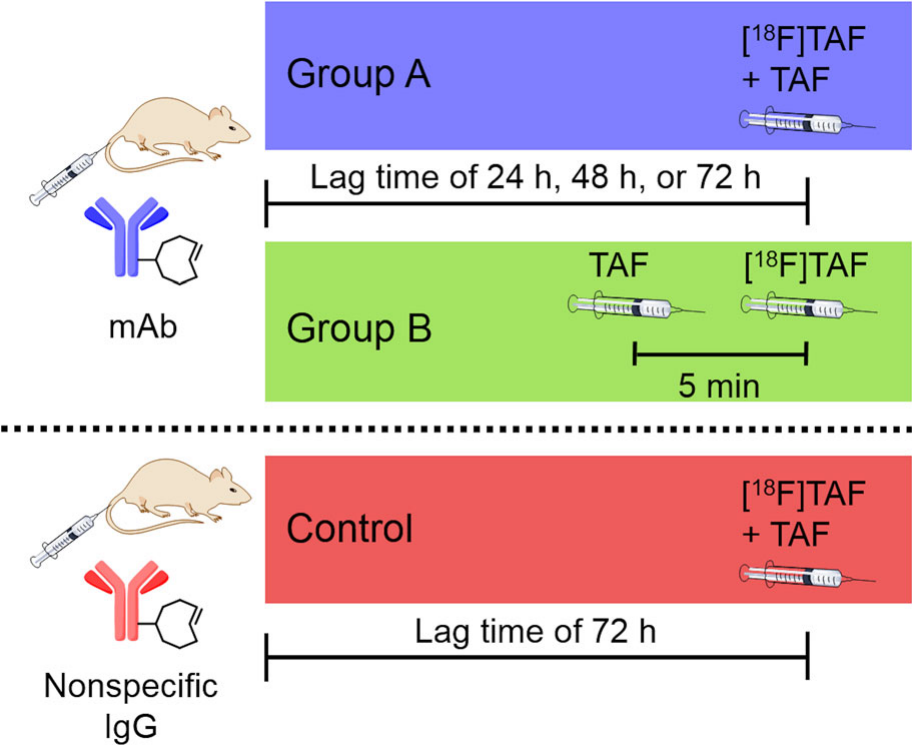
Experimental scheme for pretargeted imaging studies. Both mAb groups (A and B) and the nonspecific IgG control received the same mass of antibody via lateral tail vein injection. In the mAb pretargeting experiments, the same total molar amount of non-radioactive TAF was given to each animal after the designated lag time of 24, 48, or 72 h either by mixing it directly to the [^18^F]TAF injection and reducing its molar activity (group A, *n* = 4) or by giving the non-radioactive TAF 5 min prior to administration of [^18^F]TAF (group B, *n* = 4). The lag time of 72 h and the single administration of the tracer was chosen for the experimental setup for nonspecific IgG control (*n* = 5) corresponding to the conditions in which the mAb pretargeting was the most successful

When the [^18^F]TAF is injected intravenously, it first encounters the mAb remaining in circulation at that time point. As a result, our experiments with high molar activity (15–38 GBq/μmol) [^18^F]TAF resulted in no observable tumour uptake (results not shown). Therefore, in order to prevent all of the injected radiotracer from reacting with mAb in the blood, some non-radioactive tracer needs to be added as a carrier. We investigated the effect of added non-radioactive TAF to the biodistribution of [^18^F]TAF in two ways (Fig. 3): the same molar amount of non-radioactive TAF was given to each animal either by mixing it directly to the [^18^F]TAF injection (group A) or by giving it intravenously 5 min prior to [^18^F]TAF injection (group B).

**Fig. 4:**
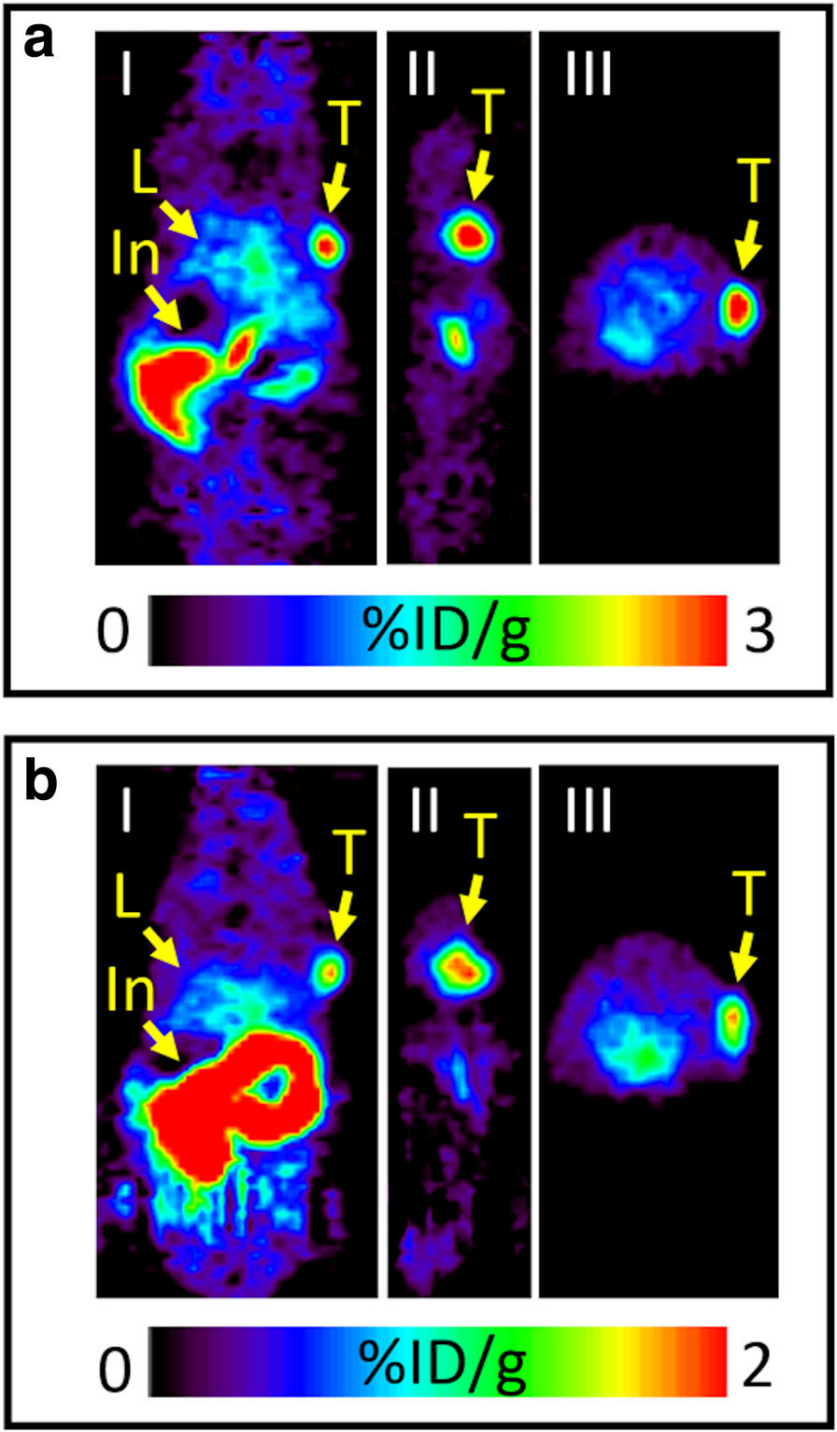
PET images of pretargeted cetuximab in A431 tumour-bearing mouse in group A (**a**) and pretargeted trastuzumab in BT-474 tumour-bearing mouse in group A (**b**). **a** 75 μg of TCO-cetuximab (3.1 nmol of TCO) was administered 72 h prior to the injection of [^18^F]TAF (4.3 ± 0.1 nmol) intravenously (*n* = 4), and **b** 20 μg of TCO-trastuzumab (0.65 nmol of TCO) was administered 72 h prior to the injection of [^18^F]TAF (1.4 ± 0.1 nmol) intravenously (*n* = 4). Coronal (I), sagittal (II), and transverse (III) planar images intersect the centre of the tumours. Arrows indicate the locations of the tumour (T), liver (L), and intestines (In)

**Fig. 5:**
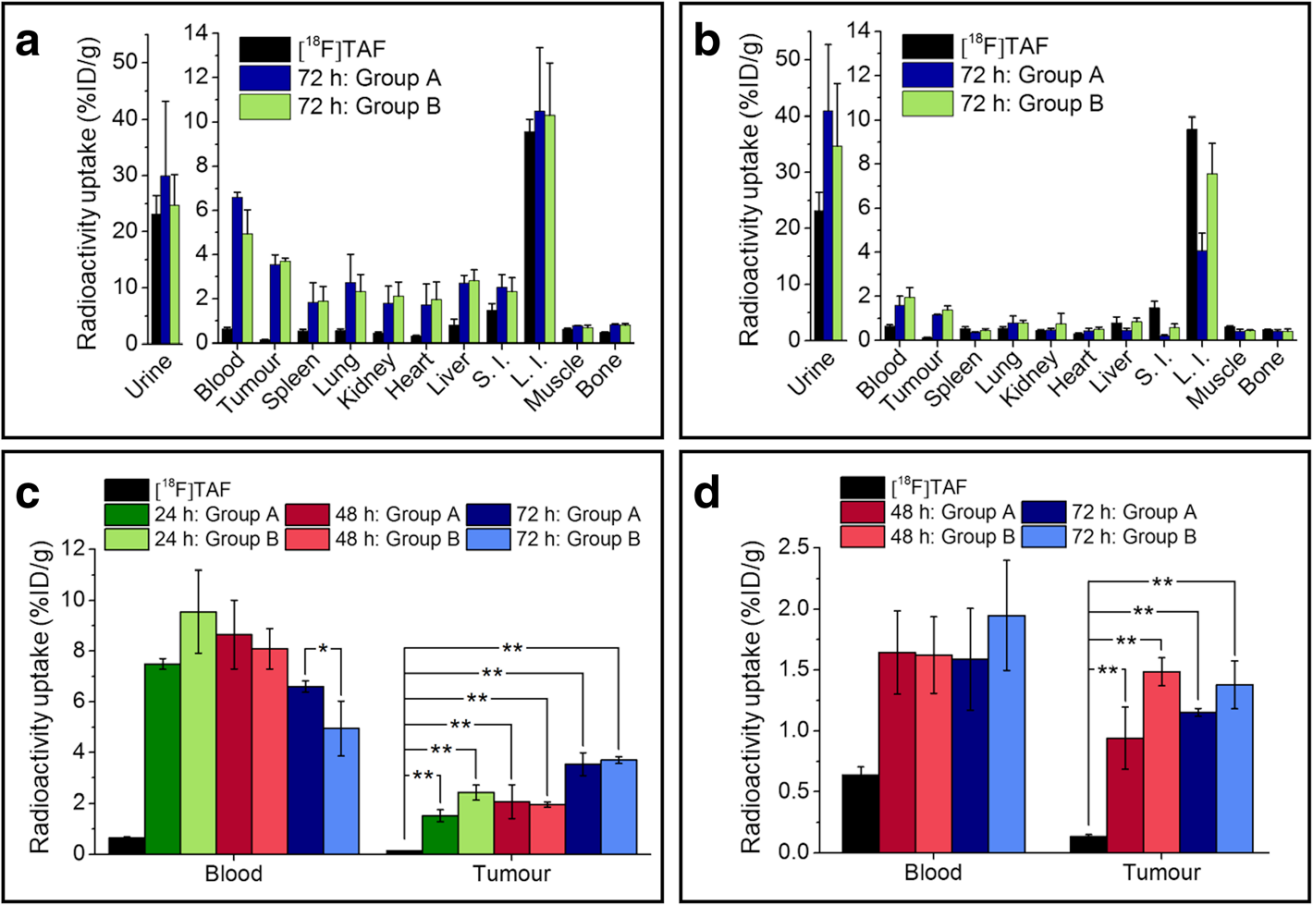
The ex vivo biodistribution of [^18^F]TAF and pretargeted cetuximab (**a**, **c**) and pretargeted trastuzumab (**b**, **d**) in groups A (*n* = 4) and B (*n* = 4). In **a** and **b**, the lag time between mAb and [^18^F]TAF injection is 72 h. All the results presented are at 4 h after [^18^F]TAF injection. The same molar amount of non-radioactive TAF was given to each animal either by adding it to the [^18^F]TAF injection (group A) or by giving it 5 min prior to [^18^F]TAF injection (group B). The values represent mean ± standard deviation. %ID/g = percentage injected dose/g. S.I. = small intestine, L.I. = large intestine. *p < 0.05 and **p < 0.005 (two-tailed paired *t* test done with IBM SPSS Statistics 22)

The in vivo IEDDA reaction was followed with PET imaging after the injection of [^18^F]TAF. For both antibodies, the tumour could be visualized from the PET images and the tumour uptake stayed constant while the background radioactivity washed out over time. Figure 4 represents PET images at 4 h after the [^18^F]TAF injection administered 72 h after the injection of mAb (group A). There was no observable difference between groups A and B in PET images (see Additional file 1: Figure S5 for PET images of group B). Results from the ex vivo biodistribution studies in mice for both pretargeted cetuximab and pretargeted trastuzumab are presented in Fig. 5 (see also Additional file 1: Tables S2 and S3). With cetuximab, the tumour uptake in pretargeted experiments was highest in the 72 h group (3.54 ± 0.45%ID/g for group A and 3.70 ± 0.13%ID/g for group B). Despite the addition of the non-radioactive carrier, some in vivo click reaction was still observed in the blood. For cetuximab, some background radioactivity was observed in the liver, lung, kidney, heart, and spleen probably due to the high blood pool of these non-target organs and clearance pathways from the liver and kidney. With cetuximab, a small decrease was observed in the radioactivity levels in the blood in group B at 72 h (group A, 6.60 ± 0.22%ID/g and group B, 4.94 ± 1.09%ID/g), which improved the tumour-to-blood ratio slightly (Table 1). This was not observed in the other experiments. For trastuzumab, the radioactivity levels in the blood and at the tumour site were the same for different lag times and in groups A and B. Because [^18^F]TAF eliminates mostly through renal excretion, the highest radioactivity levels were observed in the urine. Rather high radioactivity levels were observed in the intestines due to the hepatobiliary excretion of [^18^F]TAF.

**Table 1:** Tumour-to-tissue ratios for pretargeted cetuximab and trastuzumab at 4 h after the injection of [^18^F]TAF

Ratio	Cetuximab 24 h, group A			Cetuximab 24 h, group B			Cetuximab 48 h, group A	Cetuximab 48 h, group B	Cetuximab 72 h, group A	Cetuximab 72 h, group B
Tumour/blood	0.20 ± 0.03			0.26 ± 0.01			0.24 ± 0.06	0.24 ± 0.01	0.54 ± 0.09	0.77 ± 0.15
Tumour/liver	0.49 ± 0.08			0.76 ± 0.15			0.72 ± 0.25	0.63 ± 0.09	1.31 ± 0.01	1.34 ± 0.20
Tumour/muscle	2.12 ± 0.38			3.41 ± 0.37			2.32 ± 0.58	2.73 ± 1.17	4.54 ± 0.59	5.42 ± 0.86
							Trastuzumab 48 h group A	Trastuzumab 48 h group B	Trastuzumab 72 h group A	Trastuzumab 72 h group B
Tumour/blood							0.57 ± 0.04	0.95 ± 0.22	0.63 ± 0.14	0.73 ± 0.17
Tumour/liver							1.64 ± 0.52	2.50 ± 0.71	1.84 ± 0.38	1.63 ± 0.14
Tumour/muscle							2.18 ± 0.48	4.12 ± 0.51	2.37 ± 0.49	3.17 ± 0.56

The same molar amount of non-radioactive TAF was given to each animal either by adding it to the [^18^F]TAF injection (group A, *n* = 4) or by giving it 5 min prior to [^18^F]TAF injection (group B, *n* = 4)

### Control experiments for nonspecific uptake

^89^Zr-labelled IgG remained mainly in the blood flow during the observation time of 5 days (see Additional file 1: Figures S2 and S3 and Table S1). Due to the enhanced permeability and retention effect, some nonspecific tumour uptake of [^89^Zr]Zr-DFO-IgG was observed in A431 and BT-474 tumour models (5.64 ± 0.46%ID/g and 5.13 ± 1.22%ID/g, respectively).

**Fig. 6:**
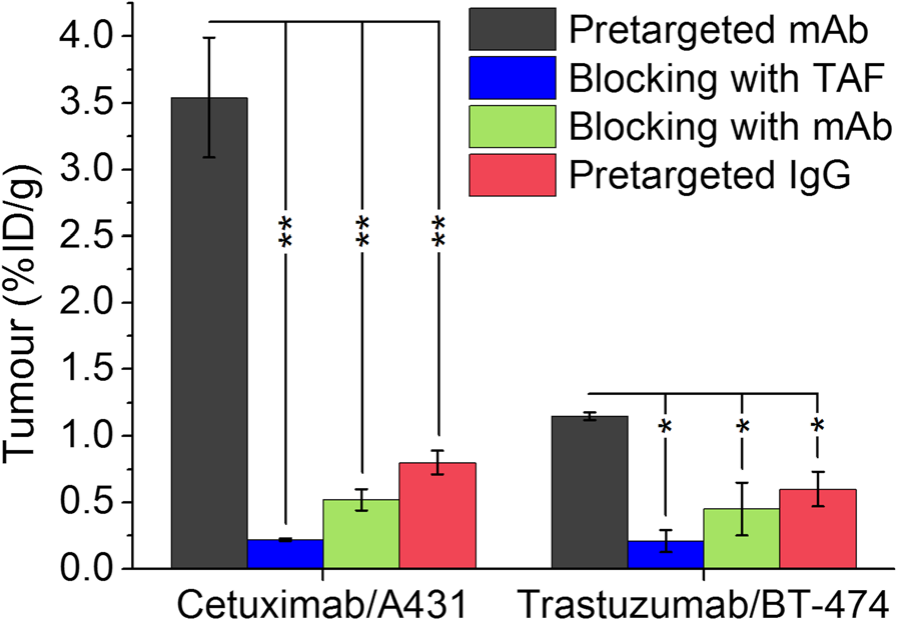
Tumour uptake values for pretargeted cetuximab and trastuzumab (group A) and nonspecific uptake controls. Nonspecific uptake controls include pretargeted TCO-cetuximab with excess of non-radioactive TAF (blue), pretargeted TCO-cetuximab with excess of mAb (green), and TCO modified immunoglobulin G pretargeted with [^18^F]TAF (red). The results presented are at 4 h after [^18^F]TAF injection. The antibody was given 72 h prior to the injection of [^18^F]TAF. The values represent mean ± standard deviation. %ID/g = percentage injected dose/g. *p < 0.05 and **p < 0.005 (two-tailed paired *t* test done with IBM SPSS Statistics 22)

Figure 6 represents the radioactivity uptake values in the tumour for pretargeted cetuximab and trastuzumab with a lag time of 72 h (group A) and control experiments for nonspecific uptake. Blocking studies with either excess of non-radioactive TAF or excess of mAb showed statistically significant decrease in the tumour uptake compared to pretargeted mAb (see Additional file 1: Table S4 and S5), thus demonstrating that [^18^F]TAF binding depends on the interaction of TCO-mAb with receptors and on the reaction between [^18^F]TAF and TCO. Radioactivity accumulated to a significantly higher degree in the tumours of mice administered TCO-mAb compared to those given TCO-IgG. PET images of the nonspecific uptake controls showed minimal radioactivity accumulation at the tumour site (Additional file 1: Figures S6, S7, and S8).

## Discussion

For both antibodies, the tumour could be visualized from the pretargeted PET images, albeit the tumour uptake of radioactivity was considerably lower than that for the ^89^Zr-labelled mAbs. This is in good agreement with the cell uptake studies (see Additional file 1: Figure S1) where we showed that the hydrophilicity of [^18^F]TAF prevents cellular internalization of the tracer alone in EGFR and HER2-positive cell lines, but in vitro pretargeting with cetuximab and trastuzumab allows for the internalization of the tracer as a result of the IEDDA reaction occurring with the mAb on the cell surface (Additional file 1: Figure S3). The degree of cell-associated radioactivity, however, is considerably lower than what is seen either with the DFO-conjugated ^89^Zr-labelled mAbs or when [^18^F]TAF is allowed to click with the TCO-modified mAb before it is applied to the cells, as illustrated by the results of the immunoreactivity assay, corroborating that the internalized fraction of the antibody remains beyond reach for this tracer.

With cetuximab, the highest tumour-to-blood ratios were achieved when the lag time between mAb and tracer injections was 72 h. This is most likely due to the lower levels of circulating unbound TCO-cetuximab in the blood flow at the later time point. An additional reason might be the downregulation of the EGF receptor induced by cetuximab [41], as the internalization rate slows down at the later time points compared to the earlier time points. This might lead to higher fraction of surface-bound cetuximab accessible for [^18^F]TAF at the later time points. With trastuzumab, no difference was seen between the lag times of 48 h and 72 h. With trastuzumab, the tumour-to-blood ratios have been reported to improve between 48 and 72 h due to a higher amount of the mAb at the tumour while the blood levels of unbound trastuzumab remain unchanged between these time points [38, 39]. This explains why we did not observe any difference in the radioactivity concentration in the blood between different antibody administration lag times with pretargeted trastuzumab. Furthermore, although there is more trastuzumab at the tumour at 72 h, the amount of non-internalized trastuzumab available for the reaction with [^18^F]TAF might in fact be the same. Hence, we did not observe increased tumour uptake at 72 h compared to 48 h.

Houghton et al. and Sharkey et al. have both showed that it is possible to pretarget an antibody that internalizes with a tracer that does not internalize [2, 42]. Our work is in accordance with this finding, but naturally, a more interesting approach would be to use a tracer that would internalize. In this case, it would be important that the unreacted radiotracer would not remain inside the cells, which could be challenging to achieve by tuning the lipophilicity of the tracer alone. This might restrict the use of internalizing radiotracers for pretargeted imaging due to the high background radioactivity in non-target organs.

Development of a “reverse” approach to Tz/TCO ligation started with the development of ^18^F-labelled TCO by Li et al. [43]. In this reverse approach, the tetrazine moiety is attached to the desired platform, e.g., antibody or nanoparticle, and the radiolabel is attached to the TCO counterpart. This approach is very appealing because the higher hydrophilicity of tetrazine compounds compared to TCO compounds may prevent hydrophobic burying of the tags to the antibody structure as reported by Rahim et al. [44] resulting in a higher effective functional loading on the surface of the platform. The reverse approach has been successfully used in pretargeted fluorescence live cell imaging, where tracer pharmacokinetics and metabolism are understandably less of a concern than in vivo [45]. However, utility of ^18^F-TCO for in vivo pretargeting is very limited [46]. ^18^F-TCO enters the brain indicating that this tracer could also internalize cells, but unfortunately, it was quickly metabolized in vivo precluding its use for pretargeting. A more stable conformationally strained TCO (s-TCO) has been used in live cell imaging and radiolabelling of biomacromolecules, but not in pretargeted experiments in live animals [47–50]. Darko et al. have developed even more stable conformationally strained TCO derivatives (d-TCO) and studied their stability and reaction kinetics [51]. The in vivo properties of the new d-TCO are very promising when considering possible internalizing tracers for pretargeting [22].

A crucial factor for the development of a successful pretargeted system is the elimination properties of the radiotracer. Renal clearance should be favoured over the hepatobiliary excretion in order to avoid unwanted, long-residing background radioactivity in the gastrointestinal tract, as high radioactivity levels in the intestines hamper the delineation of nearby organs. The pharmacokinetic and elimination profile of a tetrazine can be altered, for example by the addition of a hydrophilic linker and/or changing the chelator [10]. Comparison of the structures and biodistribution of the published tetrazines [2, 4, 9–13, 19, 20, 22] for pretargeted PET imaging shows that there are multiple factors that contribute to the pharmacokinetic profile. However, there might be one highly influencing factor, namely the introduction of positive charge to a radiopharmaceutical, which can increase the renal clearance [52, 53]. Despite the non-optimal elimination kinetics of [^18^F]TAF, our data shows that it can be used for the pretargeted PET imaging of antibodies. Given that this approach for pretargeting cetuximab and trastuzumab is intended for the imaging of tumours outside the abdominal area, the hepatobiliary excretion would not interfere with the image analysis as much as it would in orthotopic colorectal tumour models. The three hydroxyl groups in the [^18^F]TAF precursor, [^18^F]-5-fluoro-5-deoxy-ribose ([^18^F]FDR), lower the lipophilicity of the radiolabelled oxime product, which is desired for fast urinary elimination. However, [^18^F]TAF has some uptake in the gastrointestinal tract and further optimization of the pharmacokinetics of [^18^F]TAF is needed to improve the present system. The structure of [^18^F]TAF, however, can be modified by attaching almost any linker between the tetrazine and the aminooxy groups. The radiolabelling could still be done by using [^18^F]FDR as a prosthetic group, making the oxime radiolabelling approach very appealing to be used with tetrazines due to the mild reaction conditions of the glycosylation. The oxime formation with [^18^F]FDR is a high-yield reaction that can be performed in room temperature in 10 min. The optimization of the structure of [^18^F]TAF towards renal clearance, however, fell beyond the scope of this work.

Recently Knight et al. presented a new pretargeted approach called HaloTag enzyme for the pretargeting of two targets: internalizing HER2 and non-internalizing TAG-72 [18]. Although the HaloTag enzyme could be used for pretargeting TAG-72 in vivo and was capable of detecting HER2 expression in vitro, it lacked the sensitivity for in vivo pretargeting of trastuzumab. The tumour uptake in the pretargeted trastuzumab experiments did not reach values significantly higher than that in pretargeted IgG. In our experiments, while the tetrazine tracer accumulated to the tumour in higher degree in the pretargeted trastuzumab experiments than in the pretargeted IgG experiments, the radioactivity accumulation in the tumour was not nearly as high as with directly radiolabelled [^89^Zr]Zr-DFO-trastuzumab. Both pretargeting approaches clearly faced the same challenge of an internalizing target antigen rapidly localizing in the cellular compartment beyond the reach of the radiotracer, prompting the future development of internalizing bioorthogonal reagents for the pretargeted imaging of trastuzumab.

Evans et al. have pretargeted cetuximab with a ^68^Ga tracer with 23-h lag time [11]. They observed high background levels in non-target organs. In our study, we observed some unbound antibody circulating even after a lag time of 72 h between the antibody and radiotracer injections. A lucrative strategy for the optimization of pretargeted systems involving long-circulating antibodies might be the use of antibody-clearing agents [54, 55]. With these agents, it might be possible to reduce the amount of circulating TCO-antibody in the blood and improve tumour delineation. This could be beneficial in two ways: (i) to improve the in vivo IEDDA reaction yield at the tumour site and (ii) to reduce the lag time between the antibody and tracer injection so that more antibody is still on the surface of the tumour cells and available for reacting with the non-internalizing tracer. However, the synthetic development of efficient TCO-scavenging clearing agents is still in its infancy limiting wider adaptation of the technique to pretargeted PET studies at present.

## Conclusions

We have pretargeted two clinically relevant monoclonal antibodies by using ^18^F-tracer under challenging conditions, wherein the mAb is internalized and unbound mAb remains in circulation, and demonstrated that a specific, bioorthogonal IEDDA reaction takes place at the tumour despite these challenges. This demonstrates the potential of the pretargeted approach for targeting of rapidly internalizing antigens expanding the scope of bioorthogonal chemistry for the development of molecular imaging tools for a wider variety of antibodies and intracellular targets. In our case, some further optimization of the pharmacokinetics of the tracer is still needed for reduction of the background radioactivity in the excretory organs. Besides the pharmacokinetic optimization of the tetrazine tracer structure, the use of an antibody clearing agent could be beneficial in the models used in order to reduce the observed high radioactivity levels in the blood remaining even after 72 h of antibody administration. It might also make it possible to shorten the time between the antibody and tracer injections, when a higher concentration of the TCO-modified antibody still resides in the cell surface available for the in vivo IEDDA reaction.

## Additional files

Additional file 1: Supporting information contains the PET images of ^89^Zr-radiolabelled cetuximab and trastuzumab; description of the purification of mAbs, immunoreactivity testing of modified mAbs, cell uptake assay, in vitro pretargeting assay, and radiosynthesis of [^18^F]TAF; and ex vivo biodistribution results for pretargeted experiments and ^89^Zr-radiolabelled cetuximab and trastuzumab. (PDF 1832 kb)

## Abbreviations

DFO: Desferrioxamine
DMEM: Dulbecco’s modified Eagle’s medium
EGFR: Epidermal growth factor receptor
FDR: Fluorodeoxyribose
HBSS: Hank’s buffered salt solution
IEDDA: Inverse electron-demand Diels-Alder
IgG: Immunoglobulin G
mAb: Monoclonal antibody
MIP: Maximum intensity projection
MSKCC: Memorial Sloan Kettering Cancer Center
PET: Positron emission tomography
p-SCN-DFO: 1-(4-Isothiocyanatophenyl)-3-[6,17-dihydroxy-7,10,18,21-tetraoxo-27-(*N*-acetylhydroxylamino)-6,11,17,22- tetraazaheptaeicosine] thiourea
RT: Room temperature
SI: Supporting information
SPAAC: Strain-promoted alkyne azide cycloaddition
SPECT: Single-photon emission computed tomography
TCO: *Trans*-cyclooctene
