# Supplementary material for: Pretargeting of internalizing trastuzumab and cetuximab with a 18F-tetrazine tracer in xenograft models
Source: EJNMMI Res. 2017 Dec 2;7:95. doi: 10.1186/s13550-017-0344-6 (PMC5712296; doi:10.1186/s13550-017-0344-6)
Supplement: Supplementary file 1 — Supporting information contains the PET images of 89Zr-radiolabelled cetuximab and trastuzumab; description of the purification of mAbs, immunoreactivity testing of modified mAbs, cell uptake assay, in vitro pretargeting assay, and radiosynthesis of [18F]TAF; and ex vivo biodistribution results for pretargeted experiments and 89Zr-radiolabelled cetuximab and trastuzumab. (PDF 1832 kb) [file 13550_2017_344_MOESM1_ESM.pdf]

Additional file 1

Pretargeting of internalizing trastuzumab and cetuximab with a ^{18}F-tetrazine tracer in xenograft models

Outi Keinänen,[1,2] Kimberly Fung,[2,3,4] Jacob Pourat,[2] Vilma Jallinoja,[1,2] Delphine Vivier,[2,3] NagaVara Kishore Pillarsetty,[2,6] Anu J. Airaksinen,[1] Jason S. Lewis,[2,5,6,7] Brian M. Zeglis,[2,3,4] Mirkka Sarparanta[1,2,*]

[1]Department of Chemistry, Radiochemistry, University of Helsinki, P.O. Box 55, FI-00014 University of Helsinki, Finland

[2]Department of Radiology, Memorial Sloan Kettering Cancer Center, 1275 York Avenue, New York, NY 10065, USA

[3]Department of Chemistry, Hunter College and the Graduate Center of the City University of New York, 695 Park Avenue, New York, NY 10065, USA

[4]Ph.D. Program in Chemistry, Graduate Center of the City University of New York, NY, 10016, USA

[5]Program in Molecular Pharmacology, Memorial Sloan Kettering Cancer Center, 1275 York Avenue, New York, NY 10065, USA

[6]Department of Radiology, Weill Cornell Medical College, 1300 York Avenue, New York, NY 10065, USA

[7]Department of Pharmacology, Weill Cornell Medical College, 1300 York Avenue, New York, NY 10065, USA

*For correspondence contact: Mirkka Sarparanta, Department of Chemistry, Radiochemistry, University of Helsinki, P.O. Box 55, FI-00014 University of Helsinki, Finland; Email: mirkka.sarparanta@helsinki.fi

Contents

1. General Materials and Methods 3
2. Radiosynthesis of ^{18}F-tracer, [^{18}F]TAF 4
3. Cell uptake assays 4
4. Immunoreactivity assay 5
5. PET images of [^{89}Zr]Zr-DFO-Abs 6
6. *In vitro* pretargeting 7
7. PET images of group B for cetuximab and trastuzumab 8
8. PET images of the nonspecific uptake controls 9
9. *Ex vivo* biodistribution 11
10. References 16

1. General Materials and Methods

Unless otherwise noted, all reagents were purchased from commercial suppliers and used without further purification. Ultrapure water (18.2 MΩ cm^{-1}) was produced on an Elga Purelab Ultra system (Elga LCC, Woodridge, IL, USA). To make Chelex treated ultrapure water, 5 g of Chelex 100 Resin (100-200 mesh, sodium form, Bio-Rad) was added to 1 l of ultrapure water and the mixture was kept under stirring overnight. The resin was let to settle and it was removed by filtration. Chelex PBS was prepared by diluting phosphate buffered saline 10× concentrate (Sigma-Aldrich) to 1× using Chelex treated ultrapure water.

Human tumour cell lines A549 and SKOV3 were obtained from the American Tissue Culture Collection (ATCC, Manassas, VA, USA). A549 cells were cultured in F-12K medium supplemented with 10% fetal bovine serum, 2 mM L-glutamine, 1.5 g/l sodium bicarbonate, penicillin, and streptomycin. SKOV3 cells were cultured in McCoy's medium supplemented with 10% fetal bovine serum, 1.5 mM L-glutamine, 2.2 g/l sodium bicarbonate, penicillin, and streptomycin. All media was purchased from the Media Preparation Facility at MSKCC (Memorial Sloan Kettering Cancer Center, New York, NY, USA). Cells were maintained in atmosphere containing 5% CO_2 at 37 °C. Cells were harvested and passaged weekly using 0.25% trypsin/0.53 mM EDTA in HBSS without calcium or magnesium.

PD10 size-exclusion column (GE Healthcare) was used for purification of cetuximab and trastuzumab before and after TCO conjugation, purification of cetuximab before and after DFO conjugation, and purification of cetuximab and trastuzumab after ^{89}Zr-labelling. The PD10 purification was done as follows. PD10 was equilibrated by gravity flow with 15 ml of PBS pH 7.4 (in the case of purifying cetuximab after DFO modification Chelex-treated PBS pH 7.4 was used), and the mAb mixture in a volume of 500 µl was put through the column. If the volume of the mAb solution was less than 500 µl, PBS pH 7.4 was added to make the volume to 500 µl. The column was washed with 2 ml of PBS pH 7.4, and the mAb was eluted with 2 ml of PBS pH 7.4. After the PD10 purification, the mAb solution was concentrated with a 50-kDa MWCO Amicon spin filter, if necessary. The Amicon filtration also purifies the mAb solution further.

After PD10 purification, the mAb solution was purified with spin-column centrifugation (Ultracel-50, Amicon, Millipore). The solution was put on an Amicon filter. The Amicon filter was filled full with PBS pH 7.4. The liquid was spinned down with a centrifuge (4000 rpm, 10-15 min). The filter was washed twice with PBS pH 7.4 with centrifugation as above. The residual liquid containing the purified mAb was transferred to a 1.5-ml low-binding Eppendorf tube and stored at +4°C until use.

2. Radiosynthesis of ^{18}F-tracer, [^{18}F]TAF

[^{18}F]FDR-tetrazine ([^{18}F]TAF = [^{18}F]**1**) was synthesized from the aminooxy-functionalized tetrazine (**5**) and [^{18}F]-5-fluoro-5-deoxy-ribose ([^{18}F]**4**) as previously described [2].

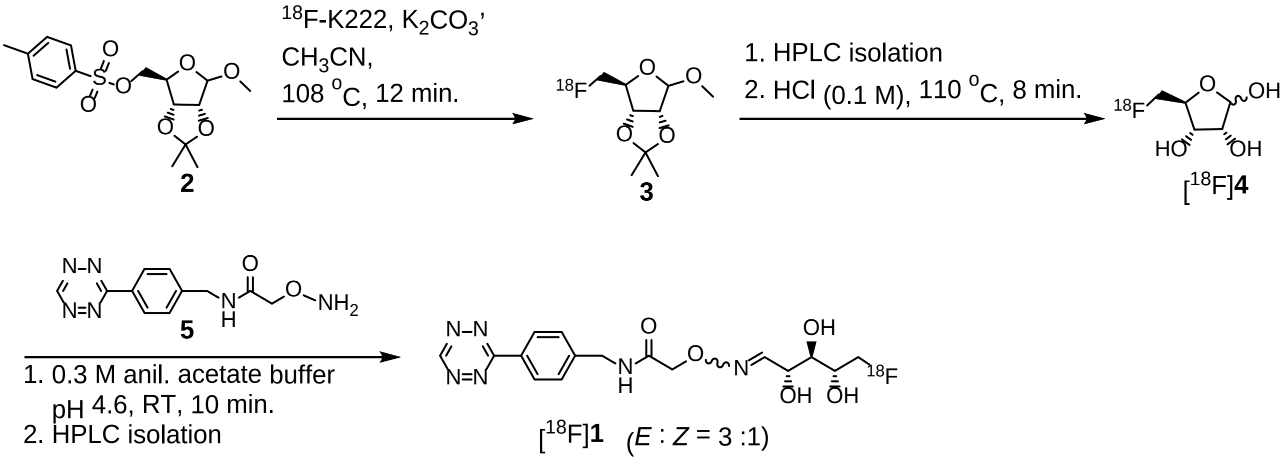

Scheme S1. Radiosynthesis of [^{18}F]TAF ([^{18}F]**1**).

3. Cell uptake assays

Cell uptake assay was performed to ensure that [^{18}F]TAF has no cell uptake. 1×10^6 BT-474 (HER2), SKOV3 (HER2) and A549 (EGFR) cells were plated per well on six-well plates. 1 ml of fresh media with 150 kBq of [^{18}F]TAF was added to each well. The cells were incubated on a shaker at 37 ˚C for 15 min, 30 min, 60 min, 120 min, and 240 min. At each time point one six-well plate per cell line was treated as follows. The plate was placed on ice and the medium was removed into gamma counting tube (*free fraction*). The cells were washed with 1 ml of ice cold PBS which was added to the gamma counting tube (*free fraction*). Then the cells were incubated twice on ice with 1 ml of 0.05 M glycine buffer pH 2.8 for 5 min. The glycine buffer was moved to gamma counting tube (*membrane-bound fraction*). The cells were washed with 1 ml of ice cold PBS which was added to the gamma counting tube (*membrane-bound fraction*). Then the cells were incubated with 1 ml of 1 M NaOH for 10 min and the NaOH was collected to gamma counting tube (*internalized fraction*). The cells were washed twice with 1 ml of ice cold PBS which were added to the gamma counting tube (*internalized fraction*). The tubes were measured with a gamma counter (Wizard^2 3”, PerkinElmer).

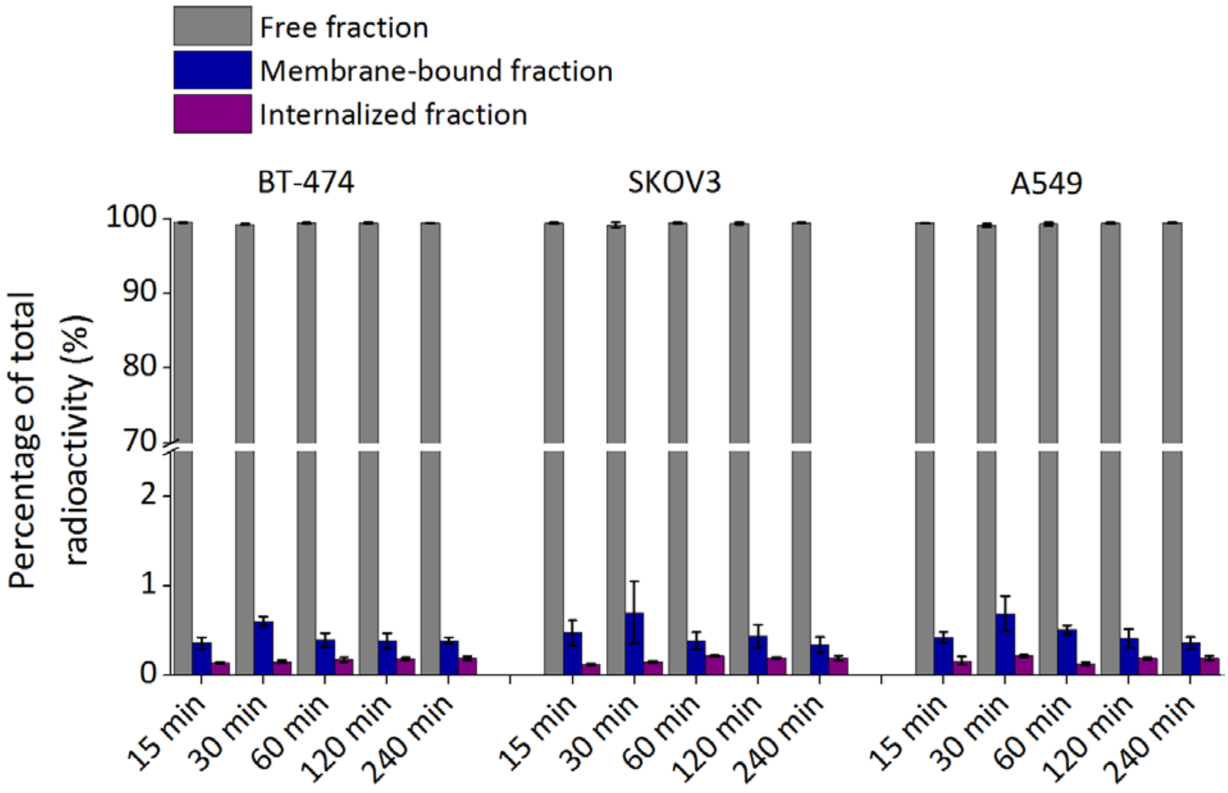

Figure S1. Cell uptake assay results for [^{18}F]TAF.

4. Immunoreactivity assay

The immunoreactivity of DFO-modified cetuximab, TCO-modified cetuximab and trastuzumab was determined as follows. DFO-cetuximab was radiolabelled with ^{89}Zr as described in Experimental procedures. TCO-mAb was conjugated with [^{18}F]TAF and purified with size-exclusion chromatography (PD10, GE Healthcare) and with spin-column centrifugation (Ultracel-50, Amicon, Millipore). 10×10^6 A431 cells for DFO-cetuximab and TCO-cetuximab, and 10×10^6 BT-474 cells for TCO-trastuzumab were put per 1.5-ml microtube (Eppendorf Protein LoBind) in five replicates per experiment. The amount of the used radiolabelled antibody ([^{89}Zr]Zr-DFO-cetuximab, [^{18}F]TAF-TCO-cetuximab, or [^{18}F]TAF-TCO-trastuzumab) was calculated to be several times less than the total amount of antibody binding sites present in the cells in the microtube. As a control, [^{18}F]TAF was incubated with the cells similar manner as DFO-cetuximab, TCO-cetuximab and TCO-trastuzumab.

The cells were centrifuged (600g, 2 min) at the bottom of the tube and the supernatant was discarded. 200 µl of ice cold cell media was added. 6 ng (0.04 pmol) of radiolabelled antibody in 20 µl of 1% BSA/1xPBS was added and the mixture was mixed well. The cells were incubated in ice for 60 min, after which they were centrifuged (600g, 2 min) and the supernatant was removed to gamma counter tube. The cells were washed with ice cold 1xPBS three times by putting 200 µl of 1xPBS into each tube, mixing the mixture, centrifuging (600g, 2 min), and collecting the supernatants to gamma counter tubes. All the supernatants and cell pellets were measured with a gamma counter (Wizard^2 3", PerkinElmer). The radioactivity bound to the cells was considered as the immunoreactive fraction of the total radioactivity. The immunoreactivities were 84.2±1.6%, 93.9±1.0% and 90.8±1.9% for DFO-cetuximab, TCO-cetuximab and TCO-trastuzumab, respectively. With [^{18}F]TAF only 1.3±0.4% of the radioactivity was bound to the cells.

5. PET images of [^{89}Zr]Zr-DFO-Abs

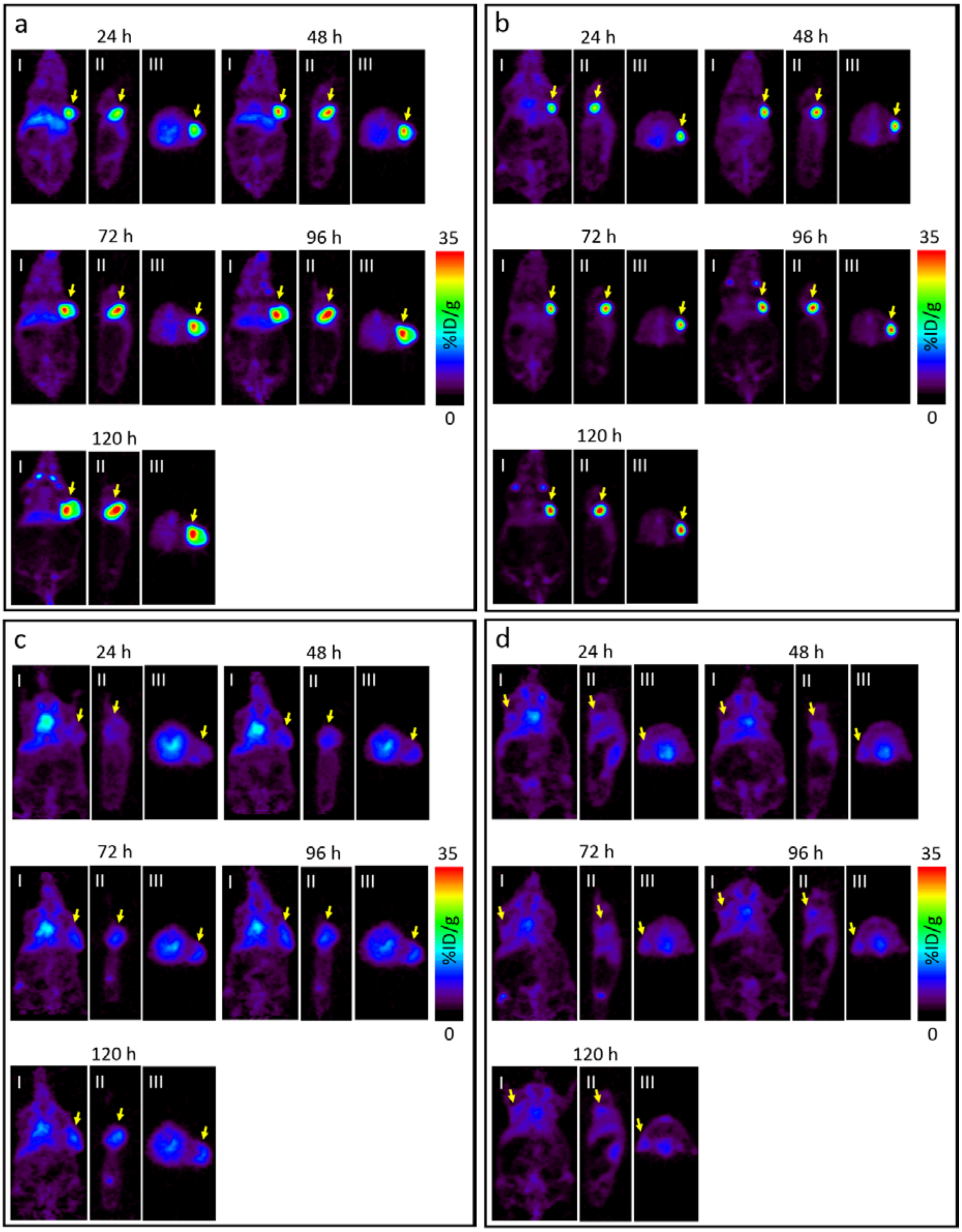

Figure S2. PET images of same representative mice a) 11.4 MBq of [^{89}Zr]Zr-DFO-cetuximab in A431 tumour bearing mouse (n=4), b) 7.3 MBq of [^{89}Zr]Zr-DFO-trastuzumab in BT-474 tumour bearing mouse (n=3), c) 9.0 MBq of [^{89}Zr]Zr-DFO-IgG in A431 tumour bearing mouse (n=3), and d) 4.0 MBq of [^{89}Zr]Zr-DFO-IgG in BT-474 tumour bearing mouse (n=3) at different time points after injection. Coronal (I), sagittal (II), and transverse (III) planar images intersect the centre of the tumours. The arrow indicates the location of the tumour

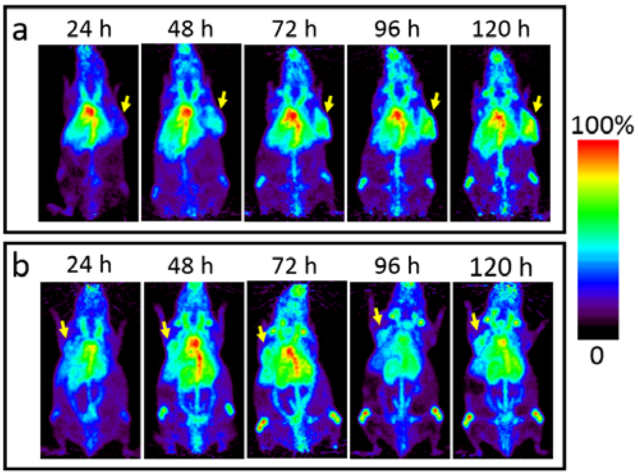

Figure S3. Maximum intensity projections (MIP) for representative mice administered with 9.0 MBq of [^{89}Zr]Zr-DFO-IgG (**a**) and 4.0 MBq of [^{89}Zr]Zr-DFO-IgG (**b**) at different time points after injection in A431(**a**) and BT-474 (**b**) tumour bearing mice (n=3), respectively. The maximum intensity projections (MIPs) are scaled to the same percentages (100%) for intensity minimum and maximum to appropriately compare the images. The arrow indicates the location of the tumour

6. *In vitro* pretargeting

Pretargeting of HER2 expressing cancer cells with trastuzumab was tested using BT-474 and SKOV3 cell lines. A549 cell line was used for pretargeting EGFR expressing cancer cells with cetuximab. The cells (1×10^6 per well on a 6-well plate) were preincubated with TCO-mAb in DMEM media for 60 min at 37 ˚C (n=6), after which the TCO-mAb media was removed. Then the cells were incubated with [^{18}F]TAF in cell media for 60 min at 37 ˚C. The free and cell-associated fractions of radioactivity were determined by automated gamma counting as described in the cell uptake assay. Three control sample sets were used. First control had 50 eq. excess of antibody incubated (60 min at 37 ˚C) with the cells before incubating with TCO-antibody. In second control 100 eq. excess of cold TAF was added to compete with the [^{18}F]TAF. In the third control, [^{18}F]TAF was incubated with cells without preincubation with TCO-antibody. *In vitro* binding of [^{18}F]TAF to TCO-antibody pre-treated cells exceeded nonspecific uptake by untreated cells over 75-fold, 20-fold, and 15-fold in BT-474, SKOV3, and A549 cells, respectively. A significant reduction of radioactivity bound to cells was observed when presaturated with antibody or under competition with large excess of cold TAF. This demonstrates that [^{18}F]TAF binding depends on the interaction of TCO-antibody with receptors and on the reaction between [^{18}F]TAF and TCO, thus confirming *in vitro* pretargeting. From the cell-associated radioactivity 76.8±1.1% (BT-474) and 61.8±0.5% (A549) was due to the internalized radioactivity.

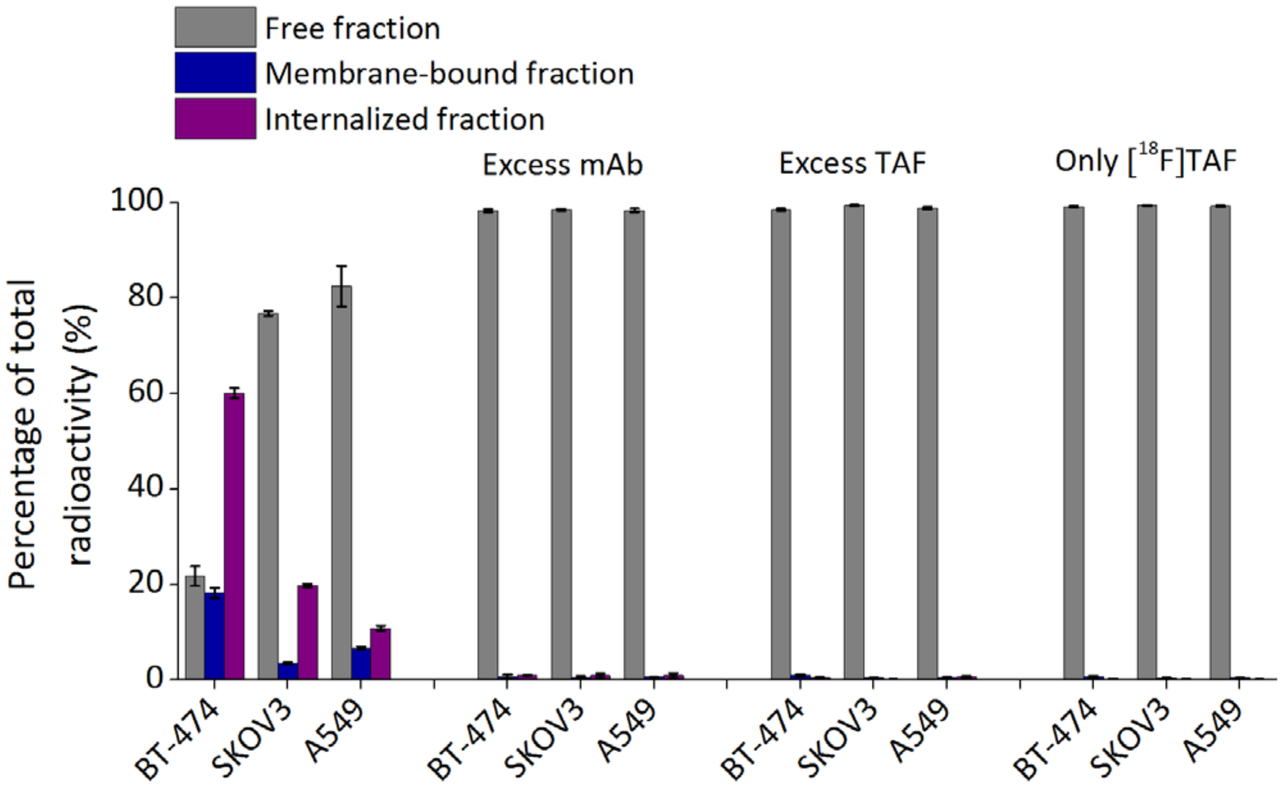

Figure S4. *In vitro* pretargeting results.

7. PET images of group B for cetuximab and trastuzumab

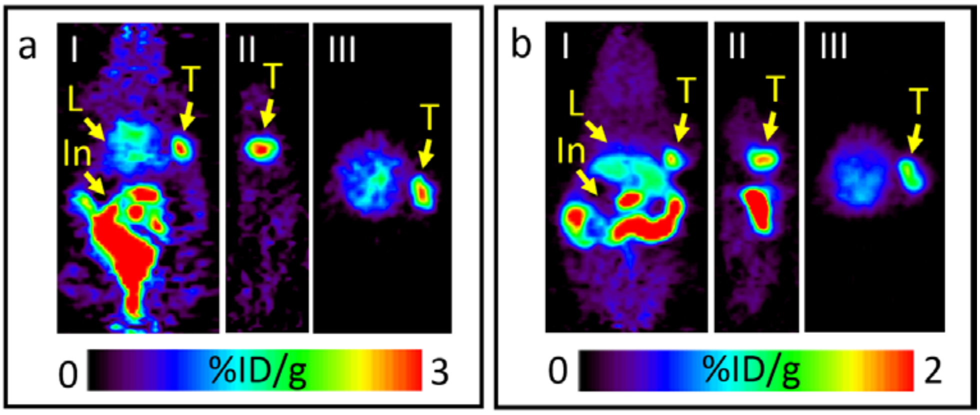

Figure S5. PET images of pretargeted cetuximab in A431 tumour bearing mouse in group B (n=4) (**a**), and pretargeted trastuzumab in BT-474 tumour bearing mouse in group B (n=4) (**b**). **a** 75 µg of TCO-cetuximab (3.1 nmol of TCO) was administered 72 h prior to the injection of TAF (3.1 nmol) followed by 5 min later injection of [^{18}F]TAF (1.2±0.1 nmol), **b** 20 µg of TCO-trastuzumab (0.65 nmol of TCO) was administered 72 h prior to the injection of TAF (0.65 nmol) followed by 5 min later injection of [^{18}F]TAF (0.6±0.1 nmol) intravenously. Coronal (I), sagittal (II), and transverse (III) planar images intersect the centre of the tumours. Arrows indicate the locations of the tumour (T), liver (L), and intestines (In)

8. PET images of the nonspecific uptake controls

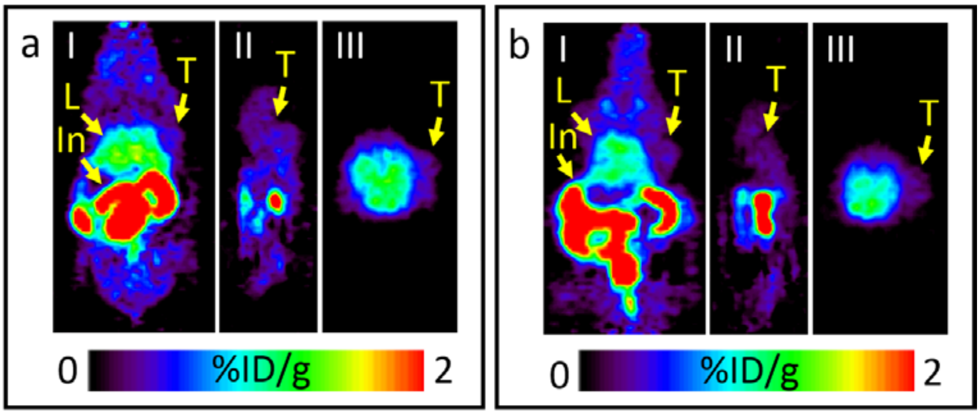

Figure S6. PET images of pretargeted cetuximab in A431 tumour bearing mouse (n=3) (**a**), and pretargeted trastuzumab in BT-474 tumour bearing mouse (n=3) (**b**) with excess of non-radioactive TAF. **a** 75 µg of TCO-cetuximab (3.1 nmol of TCO) was administered 72 h prior to the injection of [$^{18/19}$F]TAF (2.68±0.05 µmol) intravenously, **b** 20 µg of TCO-trastuzumab (0.65 nmol of TCO) was administered 72 h prior to the injection of [^{18}F]TAF (0.52±0.01 µmol) intravenously. Coronal (I), sagittal (II), and transverse (III) planar images intersect the centre of the tumours. Arrows indicate the locations of the tumour (T), liver (L), and intestines (In)

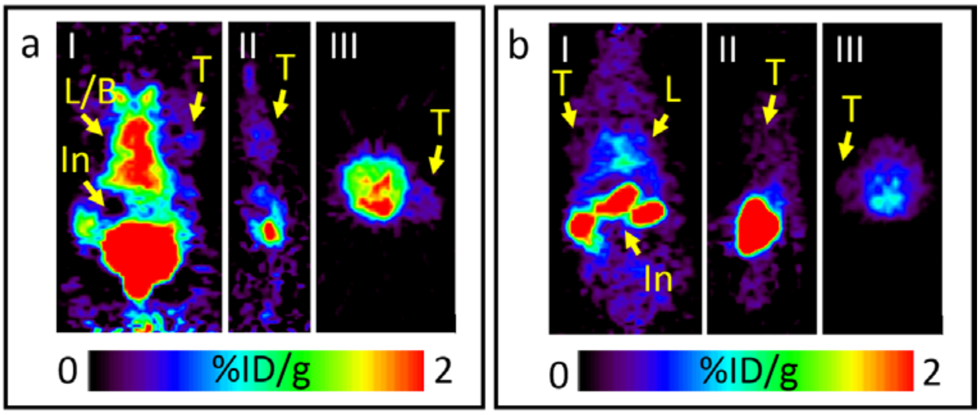

Figure S7. PET images of pretargeted TCO-cetuximab in A431 tumour bearing mouse (n=3) (**a**), and pretargeted TCO-trastuzumab in BT-474 tumour bearing mouse (n=5) (**b**) with 25-fold excess of mAb (unmodified with TCO). **a** 75 µg of TCO-cetuximab (1.3 nmol of TCO) and 1875 µg of cetuximab was administered 72 h prior to the injection of [^{18}F]TAF intravenously, **b** 20 µg of TCO-trastuzumab (0.3 nmol of TCO) and 500 µg of trastuzumab was administered 72 h prior to the injection of [^{18}F]TAF intravenously. Coronal (I), sagittal (II), and transverse (III) planar images intersect the centre of the tumours. Arrows indicate the locations of the tumour (T), liver (L), blood (B) and intestines (In)

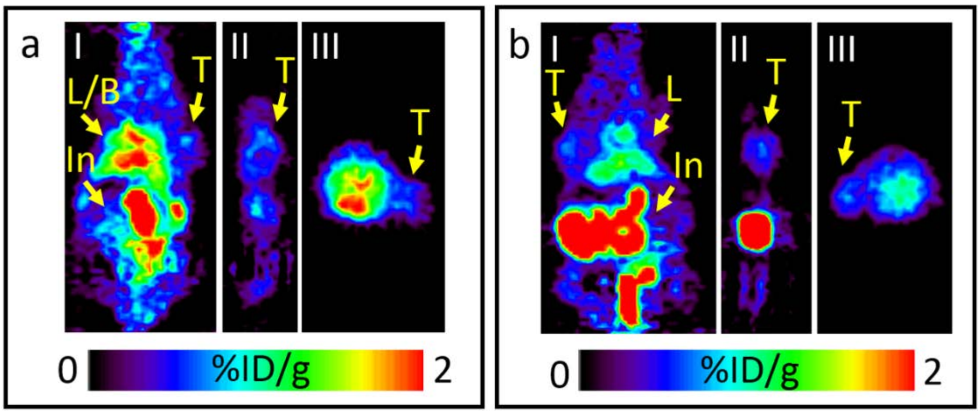

Figure S8. PET images of pretargeted TCO-IgG in A431 tumour bearing mouse (n=5) (**a**), and pretargeted TCO-IgG in BT-474 tumour bearing mouse (n=5) (**b**). **a** 75 µg of TCO-IgG (0.5 nmol, 1.2 nmol of TCO) was administered 72 h prior to the injection of [^{18}F]TAF intravenously, **b** 20 µg of TCO-IgG (0.13 nmol, 0.3 nmol of TCO) was administered 72 h prior to the injection of [^{18}F]TAF intravenously. Coronal (I), sagittal (II), and transverse (III) planar images intersect the centre of the tumours. Arrows indicate the locations of the tumour (T), liver (L), blood (B) and intestines (In)

9. *Ex vivo* biodistribution

Table S1. The *ex vivo* biodistribution of [^{89}Zr]Zr-DFO-cetuximab (n=4), [^{89}Zr]Zr-DFO-trastuzumab (n=3) and [^{89}Zr]Zr-DFO-IgG (n=3) at 120 h after injection. P-values were determined using a 2-tailed paired T-test (mAb vs. non-specific IgG) done with IBM SPSS Statistics 22 (*p<0.05 and **p<0.005).

	[^{89}Zr]Zr-DFO-cetuximab			[^{89}Zr]Zr-DFO-IgG, A431				[^{89}Zr]Zr-DFO-trastuzumab			[^{89}Zr]Zr-DFO-IgG, BT-474			
**Tissue**	%ID/g			%ID/g			p-value	%ID/g			%ID/g			p-value
**Blood**	7.99	±	0.07	9.40	±	1.16	0.1738	3.77	±	0.23	6.58	±	2.34	0.1778
**Tumour**	22.01	±	2.19	5.64	±	0.46	0.0049**	37.56	±	4.99	5.13	±	1.22	0.0034**
**Spleen**	4.93	±	0.56	6.99	±	2.12	0.2912	2.31	±	0.19	2.49	±	0.76	0.7110
**Lung**	4.23	±	0.01	5.30	±	1.15	0.2476	2.30	±	0.34	3.99	±	1.29	0.1532
**Kidney**	3.36	±	0.21	6.00	±	0.21	0.0060*	2.77	±	0.73	8.27	±	0.19	0.0037**
**Heart**	2.34	±	0.23	2.60	±	0.66	0.5647	1.43	±	0.23	2.64	±	0.76	0.1028
**Liver**	5.81	±	1.37	4.46	±	0.98	0.3505	3.48	±	1.49	4.77	±	0.75	0.3387
**Small intestines**	0.98	±	0.05	0.98	±	0.17	0.9418	0.40	±	0.04	0.99	±	0.33	0.0962
**Large intestines**	0.82	±	0.04	0.81	±	0.19	0.9608	0.48	±	0.18	0.59	±	0.14	0.5515
**Skeletal muscle**	0.66	±	0.02	0.88	±	0.07	0.0447*	0.41	±	0.01	0.73	±	0.23	0.1347
**Bone**	9.64	±	0.20	6.30	±	0.71	0.0209*	4.86	±	0.61	9.50	±	1.83	0.0289*

Table S2. The *ex vivo* biodistribution of pretargeted TCO-cetuximab. The results presented are at 4 hours after [^{18}F]TAF injection. Cetuximab was given 24 h, 48 h, or 72 h prior the injection of [^{18}F]TAF. Same amount of non-radioactive TAF was given to each animal either by adding it to the [^{18}F]TAF injection (group A, n=4) or by giving it 5 min prior to [^{18}F]TAF injection (group B, n=4). The values represent mean ± standard deviation. %ID/g = percentage injected dose/gram.

	24 h Group A	24 h Group B	48 h Group A	48 h Group B	72 h Group A	72 h Group B
**Tissue**	%ID/g	%ID/g	%ID/g	%ID/g	%ID/g	%ID/g
**Urine**	25.16 ± 9.56	29.08 ± 2.10	36.72 ± 10.40	27.70 ± 5.22	29.94 ± 13.20	24.74 ± 5.44
**Blood**	7.50 ± 0.22	9.55 ± 1.64	8.65 ± 1.35	8.09 ± 0.80	6.60 ± 0.22	4.94 ± 1.09
**Tumour**	1.52 ± 0.24	2.43 ± 0.29	2.07 ± 0.66	1.96 ± 0.10	3.54 ± 0.45	3.70 ± 0.13
**Spleen**	1.57 ± 0.11	1.66 ± 0.78	2.06 ± 0.41	1.11 ± 0.21	1.84 ± 0.89	1.89 ± 0.66
**Lung**	2.76 ± 0.25	3.19 ± 0.03	3.33 ± 0.51	1.70 ± 0.20	2.73 ± 1.28	2.32 ± 0.77
**Kidney**	1.82 ± 0.48	1.52 ± 0.03	1.75 ± 0.40	1.17 ± 0.04	1.80 ± 0.77	2.11 ± 0.64
**Heart**	1.93 ± 0.13	1.96 ± 0.21	2.51 ± 0.48	1.35 ± 0.32	1.72 ± 0.95	1.96 ± 0.80
**Liver**	3.14 ± 0.17	3.20 ± 0.27	2.87 ± 0.28	3.13 ± 0.30	2.70 ± 0.36	2.81 ± 0.49
**Small intestines**	1.21 ± 0.22	1.12 ± 0.23	1.37 ± 0.10	1.87 ± 0.39	2.52 ± 0.57	2.32 ± 0.63
**Large intestines**	10.78 ± 2.20	9.51 ± 2.93	8.75 ± 1.72	15.02 ± 1.41	10.48 ± 2.89	10.29 ± 2.37
**Skeletal muscle**	0.72 ± 0.07	0.72 ± 0.16	0.88 ± 0.13	0.78 ± 0.30	0.78 ± 0.00	0.69 ± 0.10
**Bone**	0.80 ± 0.04	0.80 ± 0.13	1.04 ± 0.21	0.84 ± 0.20	0.83 ± 0.03	0.79 ± 0.08

Table S3. The *ex vivo* biodistribution of pretargeted TCO-trastuzumab. The results presented are at 4 hours after [^{18}F]TAF injection. Trastuzumab was given 48 h, or 72 h prior the injection of [^{18}F]TAF. Same amount of non-radioactive TAF was given to each animal either by adding it to the [^{18}F]TAF injection (group A, n=4) or by giving it 5 min prior to [^{18}F]TAF injection (group B, n=4). The values represent mean ± standard deviation. %ID/g = percentage injected dose/gram.

	48 h Group A	48 h Group B	72 h Group A	72 h Group B
**Tissue**	%ID/g	%ID/g	%ID/g	%ID/g
**Urine**	35.32 ± 7.62	30.76 ± 9.56	40.91 ± 11.80	34.69 ± 11.08
**Blood**	1.64 ± 0.34	1.62 ± 0.31	1.59 ± 0.42	1.95 ± 0.45
**Tumour**	0.94 ± 0.26	1.49 ± 0.11	1.15 ± 0.03	1.38 ± 0.20
**Spleen**	0.35 ± 0.02	0.43 ± 0.10	0.36 ± 0.04	0.46 ± 0.08
**Lung**	0.71 ± 0.12	0.81 ± 0.25	0.80 ± 0.30	0.80 ± 0.11
**Kidney**	0.62 ± 0.11	0.51 ± 0.18	0.46 ± 0.08	0.75 ± 0.48
**Heart**	0.45 ± 0.01	0.44 ± 0.05	0.43 ± 0.11	0.50 ± 0.11
**Liver**	0.58 ± 0.03	0.64 ± 0.20	0.45 ± 0.11	0.85 ± 0.16
**Small intestines**	0.40 ± 0.12	0.43 ± 0.26	0.21 ± 0.05	0.58 ± 0.17
**Large intestines**	7.81 ± 1.22	7.43 ± 2.55	4.06 ± 0.80	7.57 ± 1.38
**Skeletal muscle**	0.43 ± 0.02	0.36 ± 0.04	0.42 ± 0.09	0.44 ± 0.04
**Bone**	0.36 ± 0.02	0.37 ± 0.10	0.42 ± 0.08	0.41 ± 0.11

Table S4. The *ex vivo* biodistribution of pretargeted TCO-cetuximab with lag time of 72 h (group A, n=4) and nonspecific uptake controls in A431 tumour model. These controls include [^{18}F]TAF (n=4), TCO modified immunoglobulin G pretargeted with [^{18}F]TAF (n=5), pretargeted TCO-cetuximab with excess of non-radioactive TAF (n=3) and pretargeted TCO-cetuximab with excess of cetuximab (n=3). The results presented are at 4 hours after [^{18}F]TAF injection. The antibody was given 72 h prior the injection of [^{18}F]TAF. The values represent mean ± standard deviation. %ID/g = percentage injected dose/gram. P-values were determined using a 2-tailed paired T-test (pretargeted mAb vs. [^{18}F]TAF or blocked condition) done with IBM SPSS Statistics 22 (*p<0.05 and **p<0.005).

	Pretarg. cetuximab	[^{18}F]TAF		Blocking with excess TAF		Blocking with excess cetuximab		Pretarg. IgG	
**Tissue**	%ID/g	%ID/g	p-value	%ID/g	p-value	%ID/g	p-value	%ID/g	p-value
**Blood**	6.60 ± 0.22	0.64 ± 0.07	0.0038**	0.97 ± 0.22	0.0044**	2.55 ± 0.06	0.0064**	4.15 ± 0.73	0.0201*
**Tumour**	3.54 ± 0.45	0.14 ± 0.02	0.0002**	0.22 ± 0.01	0.0030**	0.52 ± 0.08	0.0027**	0.80 ± 0.09	0.0005**
**Spleen**	1.84 ± 0.89	0.53 ± 0.08	0.0464*	0.60 ± 0.13	0.0968	0.56 ± 0.44	0.1106	0.80 ± 0.13	0.2526
**Lung**	2.73 ± 1.28	0.55 ± 0.08	0.0494*	0.69 ± 0.07	0.0538	1.14 ± 0.07	0.0915	1.38 ± 0.26	0.0851
**Kidney**	1.80 ± 0.77	0.44 ± 0.05	0.0440*	0.51 ± 0.06	0.0624	0.63 ± 0.03	0.0580	1.06 ± 0.15	0.0746
**Heart**	1.72 ± 0.95	0.31 ± 0.03	0.0543	0.32 ± 0.02	0.0667	0.61 ± 0.05	0.0988	1.13 ± 0.23	0.0896
**Liver**	2.70 ± 0.36	0.80 ± 0.27	0.0221*	0.65 ± 0.13	0.0093*	1.22 ± 0.16	0.0248*	1.76 ± 0.43	0.0416*
**Small intestines**	2.52 ± 0.57	1.47 ± 0.31	0.0113*	0.40 ± 0.10	0.0085*	0.54 ± 0.22	0.0111*	0.69 ± 0.23	0.0443*
**Large intestines**	10.48 ± 2.89	9.58 ± 0.56	0.0568	6.50 ± 0.48	0.1014	7.24 ± 1.15	0.0506	6.45 ± 2.58	0.1396
**Skeletal muscle**	0.78 ± 0.00	0.61 ± 0.05	0.0018**	0.35 ± 0.05	0.0025**	0.37 ± 0.14	0.0359*	0.57 ± 0.13	0.0413*
**Bone**	0.83 ± 0.03	0.48 ± 0.03	0.0001**	0.35 ± 0.05	0.0003**	0.47 ± 0.10	0.0197*	0.76 ± 0.12	0.5605

Table S5. The *ex vivo* biodistribution of pretargeted TCO-trastuzumab with lag time of 72 h (group A, n=4) and nonspecific uptake controls in BT-474 tumour model. These controls include [^{18}F]TAF (n=4), TCO modified immunoglobulin G pretargeted with [^{18}F]TAF (n=5), pretargeted TCO-trastuzumab with excess of non-radioactive TAF (n=3) and pretargeted TCO-trastuzumab with excess of trastuzumab (n=5). The results presented are at 4 hours after [^{18}F]TAF injection. The antibody was given 72 h prior the injection of [^{18}F]TAF. The values represent mean ± standard deviation. %ID/g = percentage injected dose/gram. P-values were determined using a 2-tailed paired T-test (pretargeted mAb vs. [^{18}F]TAF or blocked condition) done with IBM SPSS Statistics 22 (*p<0.05 and **p<0.005).

	**Pretarg. trastuzumab**	**[^{18}F]TAF**		**Blocking with excess TAF**		**Blocking with excess trastuzumab**		**Pretarg. IgG**	
**Tissue**	%ID/g	%ID/g	p-value	%ID/g	p-value	%ID/g	p-value	%ID/g	p-value
**Blood**	1.59 ± 0.42	0.64 ± 0.07	0.0678	0.81 ± 0.14	0.0943	1.30 ± 0.24	0.1032	1.41 ± 0.25	0.1809
**Tumour**	1.15 ± 0.03	0.14 ± 0.02	0.0015**	0.21 ± 0.08	0.0089*	0.45 ± 0.20	0.0116*	0.60 ± 0.13	0.0132*
**Spleen**	0.36 ± 0.04	0.53 ± 0.08	0.2045	0.41 ± 0.10	0.5077	0.37 ± 0.10	0.9409	0.80 ± 0.13	0.3487
**Lung**	0.80 ± 0.30	0.55 ± 0.08	0.1415	0.60 ± 0.19	0.3870	0.66 ± 0.15	0.1760	0.55 ± 0.12	0.0886
**Kidney**	0.46 ± 0.08	0.44 ± 0.05	0.0428*	0.40 ± 0.07	0.4923	0.35 ± 0.09	0.1331	0.64 ± 0.53	0.5259
**Heart**	0.43 ± 0.11	0.31 ± 0.03	0.0778	0.36 ± 0.03	0.3719	0.48 ± 0.07	0.2943	0.40 ± 0.07	0.3288
**Liver**	0.45 ± 0.11	0.80 ± 0.27	0.9054	0.59 ± 0.04	0.1747	0.57 ± 0.15	0.1551	0.75 ± 0.17	0.2800
**Small intestines**	0.21 ± 0.05	1.47 ± 0.31	0.0228*	0.71 ± 0.19	0.0110*	0.30 ± 0.12	0.5926	0.30 ± 0.05	0.0533
**Large intestines**	4.06 ± 0.80	9.58 ± 0.56	0.2922	5.38 ± 0.61	0.0473*	6.40 ± 1.04	0.1539	7.15 ± 0.99	0.0093*
**Skeletal muscle**	0.42 ± 0.09	0.61 ± 0.05	0.1910	0.38 ± 0.02	0.5355	0.46 ± 0.19	0.7051	0.37 ± 0.14	0.2533
**Bone**	0.42 ± 0.08	0.48 ± 0.03	0.0785	0.32 ± 0.07	0.1390	0.54 ± 0.28	0.7330	0.46 ± 0.30	0.0931

10. References

1. Holland JP, Sheh Y, Lewis JS. (2009) Standardized methods for the production of high specific-activity zirconium-89. Nucl Med Biol 36:729-39

2. Keinänen O, Li XG, Chenna NK, Lumen D, Ott J, Molthoff CF et al. (2016) A New Highly Reactive and Low Lipophilicity Fluorine-18 Labeled Tetrazine Derivative for Pretargeted PET Imaging. ACS Med Chem Lett 7:62-6
